# An analysis of trade cooperation: Central region in China and ASEAN

**DOI:** 10.1371/journal.pone.0261270

**Published:** 2021-12-22

**Authors:** Roni Bhowmik, Yuhua Zhu, Kuo Gao

**Affiliations:** 1 School of Business, Guangdong University of Foreign Studies, Guangzhou, China; 2 Department of Business Administration, Daffodil International University, Dhaka, Bangladesh; 3 School of Economics and Management, Jiujiang University, Jiangxi, China; Szechenyi Istvan University: Szechenyi Istvan Egyetem, HUNGARY

## Abstract

China-ASEAN are the two huge markets in trade world, they can bring out greater dynamism from within their economies and contribute to regional economic development. This study explores the present situation on the trade between the Central region of China and ASEAN through empirical assessment and try to find the potential effects and trade flows between them. Firstly, we analysis the trade integration index, HM index, explicit comparative advantage index, and trade complementarity index. Finally, we use the gravity model of international trade and data on 2006–2018. The bilateral trade relations between the central region and ASEAN are getting closer, but the central region has not yet become the major trade area of ASEAN countries in the Chinese market. The bilateral economic development level plays a positive role in promoting the export trade between the Central region and ASEAN, while the bilateral distance plays a negative role in difficulty. The empirical results show that trade potential between the Central region and Indonesia and the Philippines is huge, and there is still opportunity for the development of the trade potential with Thailand. The trade prospective with Malaysia, Singapore and Vietnam is limited, and new approaches need to be developed to achieve further trade cooperation.

## 1. Introduction

Since the formal establishment of the strategic partnership between China and Association of Southeast Asian Nations (ASEAN), China has become ASEAN’s largest trading partner for ten consecutive years, and ASEAN has also become China’s third largest trading partner for eight consecutive years [1]. China and six ASEAN countries have achieved zero tariff upon 90% of goods. The average tariff of China to ASEAN goods has dropped from 9.8% to 0.1%, while the average tariff of ASEAN to China goods has dropped from 12.8% to 0.6%, which directly bring the growth of bilateral trade and investment [2]. In 2017, the bilateral trade volume had exceeded USD 500 billion, and the two-way direct investment had accumulated nearly USD 200 billion. In 2018, on the occasion of the 15th anniversary of the establishment of the strategic partnership between China and ASEAN, it has been announced by China and Asian about the vision of China ASEAN strategic partnership 2030, marking that China ASEAN relations have officially entered a new era.

The Central region (six provinces under Central region in China, those are–Jiangxi, Hubei, Hunan, Anhui, Shanxi, and Henan province) of China is located in the inland of China and is in a weak position for foreign trade. For a long time, the development of foreign trade in the Central region is in relatively backward, and the trade scale of the Central provinces is far less developed than that of the Eastern provinces [3]. The Central region does not have the geographical advantages of the border provinces such as Guangxi and Yunnan, and the convenience of coastal provinces such as Guangdong and Fujian in maritime transportation. After the several issuance and implementation of the opinions of the CPC (Central Committee and the State Council), it has started promoting the rise of the Central region in 2006. From 2006 to 2010, the import and export volume of the six Central provinces had increased from 53.6 billion U.S. dollars to 387.3 billion dollars. In our study period we found that, Central region annual growth rate was higher than in China’s other regions (2005–2010 growth rate 11.8, 2010–2015 growth rate 7.4, 2015–2018 growth rate 6.6). Compare to the year of 2006, the import and export growth rate in 2007 reached to 37.7% which was 14.2% points higher than the national average growth rate. During the period, despite the impact of the global financial crisis, the total amount of import and export in the Central region declined in 2009, however, the foreign trade of Central region still maintained a rapid growth trend.

After the global financial crisis with the wave of industrial transfer in the eastern coastal areas, the State Council has issued the guideline on undertaking industrial transfer in the central and western regions. The guideline follows promoting the Central region to undertake industrial transfer in the Eastern region, promoting the development of processing trade in the Central region, and promoting the rapid growth of foreign trade in the Central region again. Gradually, the Central region has become an important growth point for China’s import and export. From 2010 to 2017, the total import and export of Central region had an average annual increase of 23.58% and this efficiency of the Central region is twice higher than that of the Western regions (average annual trade increase 11.13%). Central region has experienced the rapid development in recent years, whereas the West region have been lagging backside in terms of both productivity and international trade, only exception in Eastern coastal areas because it is the international hub of China’s export and import trade and this area accounts cover more than 50% of the national GDP [4]. These data shows that the level of economic development in Central region has increased significantly and the vitality of development has improved.

Roberts [5] has analysed trade data of China and ASEAN countries before and after the start of the free trade zone, has established a corresponding econometric model, and found that China and ASEAN are closer in terms of per capita income and demand, and the amount of trade between them is also greater. The economic and trade cooperation between China and ASEAN is advancing step by step in the increasingly close relationship between the two countries. In 2015, to promote the vision and action of the One Belt One Road (OBOR), the joint construction of the Silk Road Economic Belt and the 21st century maritime silk road has been categorized together and it has always been the main market of foreign trade in the Central region [6]. The English translation is changed from One Belt One Road (OBOR) to the Belt and Road Initiative (BRI) since 2016. Even though, BRI have their particular resource and strategy benefits, their economies being jointly complementary, for this reason, there is noteworthy possibility and space for collaboration with other countries. BRI have a chance to progress the integration and transform their economic circumstances through exploring complementarities by trade and investment China and South Asia countries [7]. To upgrade and expand the BRI business routes to Europe probably mean further opportunities for the EU- China-ASEAN trade [8]. BRI build an opportunity for interconnection, integrated Eurasian continent with China and this connectivity will exceed the trade history [9]. China pursuing lots of projects in African countries, and maximum projects under the BRI project to do help construct infrastructure, industry and connectivity across the continent but the experiences with BRI are mixed [10].

However, Chinese economy is increased and trade between China and ASEAN countries can be expected high in the years to come, because of the BRI. In future, the increase of China’s regional impact can be assisted by BRI. Therefore, the connection with BRI strategy can be effective for Central region if they want to increase the level of foreign trade then ASEAN will be an important breakthrough [11]. China’s Central region and ASEAN have great potential for economic and trade cooperation, and there is great potential for increasing cooperation. Asian is progressing gradually by implementing an economic recovery plan and it shows the possibility of building economic cooperation with China. Industries from both China and ASEAN observe the cooperation of potential Central region. Though the Central region has a certain industrial foundation but it is not satisfactory in compared with the Eastern region where the level of cooperation with ASEAN countries generally has more possibility for improvement. However, the economic and trade cooperation between the Central region and ASEAN countries has extensive prospects.

However, the total trade volume between China and ASEAN is very immense but the trade scale between the Central region and ASEAN is doing only the small portion of that because of the limitation of geographical location [4, 12]. As central government gives more priority to the Central region, we choose the Central region and ASEAN countries as the research object for the study, even though, there are two more reasons. First, the Central region and ASEAN have very important strategic position, and it help to focus on the development of foreign trade in the Central region, although, the trade cooperation between the Central region and ASEAN has a long history of more than 20 years. In the past years, the investigation was about the results of bilateral trade, the current situation of trade relationship, which industries have complementary advantages, and some factors that affect bilateral trade flows under the current trade conditions, and whether there is trade potential to continue to dig out the evidence that very little is known. Therefore, the study of trade cooperation between the Central region and ASEAN has direct practical significance. Second, the continued deepening of China-ASEAN economic and trade cooperation cannot rely solely on the southwestern advantageous provinces, but should be realized in the whole river basin. The advantaged provinces will lead to the cooperation of other provinces that do not have the advantage. The research on trade cooperation between the Central region and ASEAN can provide reference for other inland provinces that do not have typical advantages in expanding trade with ASEAN.

Researchers point out that the trade relations between China and ASEAN countries are more complementary [13, 14]. Yang & Sun [15] measure the competitiveness and complementarity of trade between China and ASEAN by using multiple indicators, and point out that China and ASEAN compete fiercely in the export of low value-added products. China and ASEAN not only have complementary advantages in resource cooperation, but also have complementary levels in economic development [16]. Jiang & Zhang [17] have been analysed intra industry trade between China and ASEAN and the results show that the level of intra industry trade between them is steadily improving. The economic and trade relations between China and ASEAN in the post crisis period results have shown new features of multi field, institutional and sub regional cooperation [18]. Some researchers have mentioned that the intra industry trade between China and ASEAN countries mainly focus on technology intensive products [19–21].

Establishment of regional integration economy can bring obvious trade effect which can be divided into trade creation effect and trade transfer effect [22]. Some scholars point out that the establishment of ASEAN-China Free Trade Area (ACFTA) will bring more trade transfer effect to China than trade creation effect [20, 23, 24]. However, some researchers have come to the opposite conclusion. Researchers mention that the establishment of ACFTA has a significant positive trade creation effect on China, and there is no trade transfer effect [25, 26]. Sheng et al. [27] makes a quantitative analysis and have found that China’s trade creation effect on ASEAN countries is extremely different in several countries, among which Vietnam, Singapore, Indonesia and other countries have significant export trade creation effect, and Indonesia and Laos have significant import trade creation effect.

Many researchers study with different provinces of China particularly the South-eastern coastal regions and ASEAN, and discuss the opportunities and prospects for the different provinces and cites ethnic areas to participate in the foreign trade of ACFTA and put forward relevant countermeasures and suggestions [28–33]. Scholars mentioned that, the competitiveness and complementarity of trade in facilities is positively correlated with the openness of the service sector in the ASEAN countries [30, 34, 35]. Cheong et al. [36] conduct an empirical analysis on the current situation and potential of trade between China and ASEAN and found that the potentiality of trade between Fujian and Malaysia and there is still room for the potential of trade with Brunei and Laos.

The Central region is located deep in the interior of China which neither have geographically borders nor seas. Therefore, compared with the South-eastern coastal region, the central region foreign trade has been lagging behind. In terms of theoretical research, researchers find that insufficient reliability is the main factor restricting the development of foreign trade in the Central region by comparing the development of foreign trade in the six central provinces and puts forward relevant policy recommendations [4, 37]. Researchers analysed that the development opportunities brought by the BRI strategy and mention that Central region is less open to the outside than other coastal open areas [38, 39]. They suggest that encouraging local enterprises to maintain stable and friendly relations with the other regions in China, they can make policy of less “go out” for local firm and more actively invite the others “please come in” which is regional international trade strategy. This will inevitably lead to competition among provinces in the ASEAN market. Under the pressure of international and domestic dual competition, the products in the central region must be more competitive if they want to stand out.

In terms of empirical analysis, the relationship between foreign trade and foreign direct investment in the major provinces based on panel data, the results show that there is a correlation between them and they promote each other [40]. Tao et al. [41] use the technology spillover model of export trade and have found that export trade has a direct effect on the economic growth of central China mainly through the improvement of its own factor productivity. Peng et al. [42] conduct the Douglas production function and analysis show that the export of the Central region as a whole play a role in promoting economic growth, but the contribution of the export trade level of some provinces to economic growth is quite different. Zhang et al. [43] examine the grey relation analysis method in the grey system and analyse the actual effect of foreign trade in the Central region of China. Performing in-depth research on the pattern and structure of foreign trade in the Central region from two aspects of spatial pattern and commodity structure [44].

Trade potential is generally a measure of the "potential trade level" between two countries and regions or multiple countries and regions. The most widely used analysis of trade potential is the gravity model and is applied to the field of international trade by economists Tinbergen [45] and Pöyhönen [46] in 1960s. There are two types of trade gravity models, one is the traditional gravity model and the extended gravity model is derived from this model. Recently a few studies have examined the trade gravity model to discuss the trade relations between China and Asian countries [47–52]. Through the traditional gravity model, the bilateral agricultural trade flows have been studied between China and ASEAN countries [19]. Zhang et al. [53] use the trade gravity model to discuss the trade relations between China, Japan and South Korea, and predict the trade potential of the three countries. An empirically study the trade potential between China and the five Central Asian countries with the help of the trade complementarity index and gravity model [54]. Zhang [55] uses the expanded gravity model to study the trade potential of China and Russia and gives some specific policy recommendations for promoting the development of trade between China and Russia. Researchers analyse those factors that affects the export trade of goods between China and the countries along the BRI with an extended gravity model, and they verify the huge trade potential between Asian and European countries along the BRI [56, 57]. Some scholars use the stochastic frontier gravity model to study the trade potential and trade efficiency of the main countries on the BRI [58–61].

The research on the economic and trade cooperation between China and ASEAN has always been the focus of academic circles. However, most of the existing studies focus on the perspective of China as a whole, and there are few studies on the economic and trade exchanges with ASEAN countries from a regional perspective. The Central region has neither the geographical advantages as other provinces but there is a major reason that the paper focuses on the Central region as ASEAN is very important for the strategic position of the central region, which is the key to the development of foreign trade in the central region.

Based on this background, this paper will start from the current situation of trade cooperation between the Central region and ASEAN. To analyse the bilateral trade relations and structure, we use a variety of trade indexes. On the basis of measuring the competitiveness and complementarity of bilateral trade products, we will find out the products with complementary advantages. Through the establishment of gravity model, the trade potential between the Central region and ASEAN countries is calculated and it will be analysed more deeply. Finally, combining the actual situation of the Central region and the analysis results, the paper puts forward specific conclusion for the further deepening of trade cooperation between the Central region and ASEAN.

There are five chapters in this paper. The contents of each chapter are as follows; the first chapter is an introduction; the second chapter methodology and data. The third chapter is the evaluation of trade relations based on the trade cooperation index, HM index, dominant competitive advantage index and trade cooperation index, the trade relations between the Central region and ASEAN are analysed quantitatively. The fourth chapter empirical analysis makes a quantitative study on the trade potential between the Central region and ASEAN countries by introducing the expanded trade gravity model. The chapter five provides conclusion part on the basis of the previous chapters.

## 2. Method and data

### 2.1 Methodology

Based on the research objective, we will analyse the current situation of trade between the Central region and ASEAN countries and this paper will evaluates three aspects of bilateral trade volume, country structure, and commodity structure through the trade integration index and HM index. It will also analyse the degree of trade closeness and dependence between the central region and ASEAN countries, and conducted an in-depth study on the competitiveness and complementarity of bilateral trade products based on the HS international classification. Finally, the core methodology applied in this study is gravity model, through the establishment of a gravity model, the trade potential between the Central region and ASEAN countries will be estimated, and the trade potential status of the central region in ASEAN countries will be analysed.

#### 2.1.1 Trade gravity model

The earliest use of it to study international trade issues has been done by the economists Tinbergen (1962) and Pöyhönen (1963). The gravity model of trade predicts that the bilateral trade volume between two countries or two regions is directly proportional to the total economic volume between the two countries and inversely proportional to the spatial distance between the two countries. The basic form of the trade gravity model is:

$$X_{ij}=A(Y_{i}Y_{j})/D_{ij}$$ (1)

In the formula, *X*_*ij*_ represents the bilateral trade volume between country *i* (exporting country) and country *j* (importing country); *A* is a constant term; *Y*_*i*_ represents the gross domestic product of country *i*; *Y*_*j*_ represents the gross domestic product of country *j*; *D*_*ij*_ represents the cost of trade and transportation distance between country *i* and country *j* is usually expressed by the distance between the capitals or economic centers of the two countries. Convert the natural logarithm to the linear form on both sides of the formula:

$${InX}_{ij}=\alpha_{0}+\alpha_{1}{InY}_{i}+\alpha_{2}{InY}_{j}+\alpha_{3}{InD}_{ij}+\mu_{ij}$$ (2)

In the formula, *α*_0_, *α*_1_, *α*_2_, and *α*_3_ are all regression coefficients, *μ*_*ij*_ which are standard random error terms, and the meanings of the remaining variables are the same as the above formula.

For the calculation of bilateral trade flows and potential, the application of the expanded trade gravity model has been more used. The new model introduces more explanatory variables into the original gravity model to improve its explanatory power and persuasion. From the perspective of variable classification, the newly introduced variables can be divided into two categories: one is endogenous variables, such as population, FDI inflows, tariff rates, real exchange rates, inflation, etc.; the other is dummy variables, such as whether to sign an FTA, whether to join the WTO, whether to be an APEC member, etc. The introduction of these new explanatory variables makes the traditional trade gravity model more accurate and reasonable in explaining international trade issues, and is more applicable. Therefore, domestic and foreign scholars also prefer to use the expanded trade gravity model.

*2*.*1*.*1*.*1 Model construction and variable description*. Based on the theoretical basis of the trade gravity model, this paper draws on previous research methods of trade potential and uses the expanded gravity model to conduct an empirical analysis of the trade potential between the Central region and ASEAN countries. Considering the possibility of data acquisition; on the basis of the two explanatory variables of traditional gravity model economic scale and distance; only the endogenous explanatory variable of population size is added, and whether it has a common boundary with China and whether it signs freedom, these two dummy variables are trade agreements. The model is finally set to:

$${InEXP}_{ij}=\alpha_{0}+\alpha_{1}{InGDP}_{i}+\alpha_{2}{InGDP}_{j}+\alpha_{3}{InDIST}_{ij}+\alpha_{4}{InPOP}_{i}+\alpha_{5}{InPOP}_{j}+\alpha_{6}{FTA}_{ij}+\alpha_{7}{Border}_{ij}+\mu_{ij}$$ (3)

In the formula, *i* represents the central region, *j* represents the trading country, *α*_0_ is the constant term, *α*_1_, *α*_2_, *α*_3_, *α*_4_, *α*_5_, *α*_6_, *α*_7_ are the correlation coefficients of each explanatory variable, and *μ*_*ij*_ are the standard random error terms. The other variables are explained as follows:

- *EXP*_*ij*_ is the explanatory variable, which represents the export volume of the Central region and various trading countries.
- *GDP*_*i*_ and *GDP*_*j*_ are explanatory variables used to reflect the economic development level and market size of the Central region and various trading countries. The higher the GDP, the greater the total economic volume of a country, and the higher the demand for trade. The size of GDP directly determines the size of bilateral trade, and there is a positive correlation between those two.
- *DIST*_*ij*_ is an explanatory variable used to measure the cost of trade and transportation between the Central region and various countries. The Central region is deep inland China, the trade and transportation methods with countries mostly rely on the sea and land multimodal transport, and Wuhan is interlaced by land, sea and air, and the transportation is convenient. The distance from Wuhan to the capitals of various countries is calculated using Google earth software, and the unit is kilometers; whether it is with China.
- *POP*_*i*_ and *POP*_*j*_ represent the population size of the Central region and various trading countries, and are used as explanatory variables to measure the size of bilateral potential markets. The increase in the number of people is directly reflected in the increase in demand for trade, which directly promotes the bilateral trade volume, and thus has a positive correlation.
- *FTA*_*ij*_ is a dummy variable and represents the formal signing of a free trade agreement between China and various trading countries. In the observation sample of this paper, in addition to the six ASEAN countries, South Korea and Australia are the countries that have signed free trade agreements with China. Among them, the China-South Korea and China-Australia free trade agreements are signed in 2015 and the ACFTA is formally established in 2010. Based on the time of signing, if the two parties sign a free trade agreement, *FTA*_*ij*_ takes 1; otherwise, *FTA*_*ij*_ takes 0.
- *Border*_*ij*_ is also a dummy variable indicating whether each trading country borders China. If it is bordered by China, *Border*_*ij*_ takes the value 1; if it is not bordered, it takes the value 0.

#### 2.1.2 Trade integration index

The concept of trade integration is first put forward by American economist Brown in 1947. In practical application, trade intensity index refers to the proportion of a country’s exports to a trading partner country in the total exports of that country, and the proportion of the total imports of that trading partner country in the total imports of other countries. The calculation formula can be expressed as follows:

$${TII}_{ij}=\frac{E_{ij}/E_{i}}{I_{j}/I_{w}}$$ (4)

In the formula: *TII*_*ij*_ represents the trade integration index of country *i* to country *j*; *E*_*ij*_ represents the total export between country *i* and country *j*; *E*_*i*_ represents the total export of country *i*; *E*_*j*_ represents the total import of country *j*; *I*_*w*_ represents the total import of the world. If *TII*_*ij*_>1 indicates that the two countries have close trade relations, the two countries are important export markets for each other; if *TII*_*ij*_<1, it indicates that the trade relationship between the two countries is relatively alienated.

#### 2.1.3 HM index

The Hubness Measurement index (HM index) refers to the center-periphery dependence index, which is proposed by Baldwin (2003) [62] to measure the degree of dependence on mutual trade between economies.


$${HM}_{ij}=\frac{E_{ij}}{E_{i}}\times (1-\frac{I_{ij}}{I_{i}})\times 100\%$$ (5)

In the formula, *E*_*ij*_ represents the export value from country *i* to country *j*, *E*_*i*_ represents the total export value from country *i*, and the ratio between the two represents the proportion of exports from country *i* and country *j* to the total exports from country *i*; The import value of *I*_*ij*_ represents the total import and export value of country *i* to country *j*, and *I*_*i*_ the ratio between the two represents the proportion of the imports of country *i* and country *j* in the total imports of country *i*. The HM index is mainly used to measure the symmetry of the dependence of the foreign trade export of country *i* on the market of country *j*.

#### 2.1.4 Comparative advantage index

The Comparative Advantage Index (RCA Index) is proposed by Balassa (1989) [63]. It is used to measure the competitiveness of a certain product or industry in the international market. The formula is:

$$RCA=\frac{X_{a}^{i}/X_{a}}{X_{w}^{i}/X_{w}}$$ (6)

In the formula, $X_{a}^{i}$ represents the export value of the category *i* commodity in the specific commodity collection in *a* region, *X*_*a*_ is the total export value of the particular commodity collection in the *a* region, $X_{w}^{i}$ represents the export value of the category *i* commodity in the specific commodity collection in the world, *X*_*w*_ represents the total export value of a specific set of commodities worldwide.

#### 2.1.5 Trade complementarity index

The Trade Complementarity Index has been put forward by Peter Drysdale in 1967 and used to measure the degree of matching between the exports of one country and the imports of another country between the two countries. Yu (2003) [64] further has proposed the method of comprehensive trade complementarity index on the basis of trade complementarity index. The formula of this index is:

$$C_{ij}=\sum \left[C_{ij}^{k}\times (\frac{W_{k}}{W})\right]$$

$$C_{ij}^{k}={RCA}_{xi}^{k}\times {RCA}_{mj}^{k}$$

$${RCA}_{xi}^{k}=\frac{X_{i}^{k}/X_{i}}{X_{w}^{k}/X_{w}}$$

$${RCA}_{mj}^{k}=\frac{M_{j}^{k}/M_{j}}{M_{w}^{k}/M_{w}}$$ (7)

Among them, *i* and *j* represent country *i* and country *j* respectively, $X_{i}^{k}$ represents country *i*′*s* export value in category *k* products, *X*_*i*_ is country *i*′*s* total export value, $X_{w}^{k}$ represents the world’s export value of category *k* products, *X*_*w*_ represents the world. For the total export value, $M_{j}^{k}$ represents the import value of category *j* products in country *j*, *M*_*j*_ represents the total import value of country *j*, $M_{w}^{k}$ represents the import value of category *k* products worldwide, and *M*_*w*_ represents the world’s total import value. *C*_*ij*_ represents the overall trade complementarity of country *i* and country *j*. The larger *C*_*ij*_ indicates the stronger trade complementarity between the two countries, $C_{ij}^{k}$ indicates the trade complementarity index between country *i*′*s* exports and country *j*′*s* imports in *k*−type products. ${RCA}_{xi}^{k}$ represents the comparative advantage of trade in category *k* products of country *i* measured by exports, and ${RCA}_{mj}^{k}$ represents the comparative advantage of trade in category *k* products of country *j* measured by imports.

Generally, the larger the ${RCA}_{xi}^{k}$, the greater the proportion of *k*−type products in the total export of country *i*, indicating that country *i* has a comparative advantage in the export of *k*−type products; when the greater ${RCA}_{mj}^{k}$, it indicates that *k*−type products account for the greater the proportion of total imports, the most important it is that country *j* has a comparative advantage in importing *k* products. If country *i* has a significant export comparative advantage in product *k*, that is, ${RCA}_{xi}^{k}$ is large, and country *j* has a significant import comparative advantage in product *k*, that is, ${RCA}_{mj}^{k}$ is large, then $C_{ij}^{k}$ is large, indicating that country *i*′*s* export and country *j*′*s* import *k* products are complementary. In the case where there are multiple types of product trade, the *C*_*ij*_ of the product trade between country *i* and country *j* can be calculated by weighting according to the proportion of the product $C_{ij}^{k}$ of *k* products and the world trade volume of *k* products *W*_*k*_ in the total trade volume *W* of all products in the world. When *C*_*ij*_>1, it indicates that the relative export share of the products of country *i* and the relative import share of the products of country *j* are highly matched. The trade in the products of the two countries is complementary, and the larger *C*_*ij*_ indicates that the trade in products of country *i* and country *j* the stronger the complementarity.

### 2.2 Data source

The trade data between the six central provinces and the ten ASEAN countries is difficult to obtain completely. This paper expands the sample size and selects eleven countries as Indonesia, Malaysia, Singapore, the Philippines, Thailand, Vietnam, Japan, South Korea, the United States, Germany and Australia as observation samples, taking 2005–2017 as the observation sample time interval, a total of 143 samples. The original six ASEAN countries are the main target market countries for foreign trade in the Central region which account for a large proportion of the Central region’s foreign trade and are relatively representative. The trade data of the Central region and countries are obtained from the total of the six provinces in the Central region. They are all derived from the 2006–2018 Statistical Yearbook of each province in the Central region and the official website of the customs. The GDP and population of the Central region come from the "Statistical Yearbook" of each province, and the GDP and population of each trading country come from the World Bank and WDI.

## 3. Evaluation of trade relations

From the analysis of the basic trade between the Central region and ASEAN, we can see that the trade relations between them is developed rapidly in the past ten years. However, the bilateral trade volume alone cannot fully explain the trade relationship between the Central region and ASEAN. This section will analyse the trade relationship between the Central region and ASEAN through the four statistical indicators of trade combination, HM index, dominant comparative advantage index and trade complementarity index.

### 3.1 Evaluation based on trade integration index

The Central region and ASEAN countries have high trade complementarity, so in theory there should be relatively large potential for trade cooperation. Therefore, it is necessary to discuss whether the Central region and ASEAN countries have a high degree of trade closeness and significance.

Table 1, shows that the index of trade integration between the Central region and ASEAN is always greater than 1 from 2005 to 2017, and after 2008, the index of trade integration between the Central region and ASEAN has increased significantly. Since China has been joined after the WTO, the Central region and ASEAN have maintained close trade ties, and continuous the increase of trade volume between them. Bhowmik & Nhoung [65] mention that trade creation and trade diversion effects depend on the trade agreement. So, the fact shows that during this period, the central region’s exports to ASEAN have been increased from US $ 20.23 billion to US $ 191.57 billion, and its percentage of exports from the Central region is also increased from 8.28% to 11.02%. In comparison, ASEAN’s trade integration index for the Central region is relatively low, which is less than 1 before 2012, only 0.685 in 2005, and remained above 1 after 2012. This shows that the trade relationship between ASEAN and the Central region is relatively alienated in the early stage, but with the successive implementation of the ACFTA and OBOR strategy, the trade relationship between ASEAN and the Central region has been significantly strengthened, and trade links have become getting closer.

**Table 1:** Bilateral trade integration index between Central region and ASEAN.

Years	2005	2006	2007	2008	2009	2010	2011	2012	2013	2014	2015	2016	2017
**Central region’s trade integration with ASEAN**	1.501	1.494	1.304	1.592	2.104	1.888	1.748	1.770	2.058	1.977	1.960	1.721	1.539
**ASEAN’s trade integration with Central region**	0.685	0.818	0.866	0.705	0.706	0.789	0.908	1.171	1.202	1.362	1.430	1.199	1.220

### 3.2 Evaluation based on HM index

The HM index is mainly used to measure the symmetry of the dependence of the foreign trade export of country *i* on the market of country *j*. The value range of the index is [0, 1], the closer to 0, the lower the degree of dependence of one country on the market of another country; on the contrary, the closer to 1, the greater the degree of dependence of the market of one country on the market of another country.

Tables 2 and 3 are the HM index data and it can be seen from the data comparison of the two tables:

**Table 2:** Central region’s HM index to major ASEAN countries from 2005 to 2017 (%).

Years	Indonesia	Malaysia	Singapore	Thailand	Vietnam	Philippines	Six ASEAN countries	Ten ASEAN countries
2005	1.37	1.39	1.49	1.72	1.12	0.60	7.37	7.86
2006	1.21	0.97	2.04	1.39	1.37	0.57	7.20	7.75
2007	1.39	1.15	1.80	1.33	1.81	0.60	7.69	7.87
2008	1.53	1.28	1.89	1.44	1.92	0.67	8.42	8.83
2009	1.72	2.41	2.23	1.65	2.41	0.77	10.78	11.73
2010	1.87	2.16	1.52	1.75	2.80	0.80	10.41	11.19
2011	1.85	2.03	1.72	1.46	2.53	0.83	9.89	10.43
2012	1.96	2.94	2.02	1.67	1.37	0.86	10.09	10.90
2013	2.29	3.29	2.25	1.86	2.06	0.94	11.84	12.69
2014	2.11	2.31	2.25	1.65	2.97	0.88	11.40	11.82
2015	1.69	2.39	2.53	1.71	2.77	0.98	11.29	11.76
2016	1.53	2.05	2.12	1.67	2.73	1.05	10.39	10.79
2017	1.63	1.81	1.49	1.62	3.05	1.15	9.99	10.01

**Sources of data:** Statistical Yearbooks of Central Provinces, National Research Network, United Nations Commodity Trade Database, ASEAN Statistical Yearbook.

**Table 3:** HM index of major ASEAN countries to the Central region from 2005 to 2017 (%).

Years	Indonesia	Malaysia	Singapore	Thailand	Vietnam	Philippines	Six ASEAN countries	Ten ASEAN countries
2005	0.36	0.11	0.04	0.15	0.32	0.04	0.13	0.13
2006	0.47	0.15	0.04	0.18	0.06	0.10	0.15	0.14
2007	0.50	0.29	0.05	0.21	0.08	0.30	0.21	0.20
2008	0.39	0.26	0.05	0.19	0.04	0.25	0.17	0.17
2009	0.47	0.23	0.06	0.22	0.10	0.33	0.20	0.20
2010	0.59	0.37	0.06	0.27	0.08	0.60	0.27	0.28
2011	0.69	0.51	0.08	0.32	0.15	0.74	0.34	0.35
2012	0.67	0.64	0.12	0.35	1.04	1.07	0.47	0.46
2013	0.84	0.65	0.17	0.43	0.92	1.01	0.53	0.52
2014	0.57	0.69	0.19	0.53	0.90	1.11	0.53	0.64
2015	0.41	0.89	0.27	0.58	1.03	1.14	0.61	0.76
2016	0.68	0.65	0.33	0.42	1.07	0.95	0.60	0.62
2017	0.69	0.75	0.32	0.51	1.34	0.87	0.68	0.69

**Sources of data:** Statistical Yearbooks of Central Provinces, National Research Network, United Nations Commodity Trade Database, ASEAN Statistical Yearbook.

Firstly, from 2005 to 2017, the HM index of ASEAN countries in the Central region shows an overall upward trend. The HM index of the six ASEAN countries increases from 7.37% to 11.84% when it is the highest, and the HM index of the ten ASEAN countries increases from 7.86% to 12.69% at the highest, which shows that the Central region’s trade dependence on ASEAN countries which is generally increasing. The result is also in agreement with the findings of previous few scholars’ studies namely [41, 56, 64]. From a country perspective, the Central region is dependent heavily on Vietnam of ASEAN, followed by Malaysia and Singapore.

Secondly, compared with the HM index of the Central region to ASEAN countries, the overall HM index of ASEAN countries to the Central region is smaller, with an average of only 0.44, which shows that the trade dependence of ASEAN countries on the Central region is generally less than that of the Central region on the ASEAN countries. It reflects that the Central region has not yet grown into a major trading area for ASEAN countries in the Chinese market.

Finally, although the HM index of bilateral trade is constantly growing, they are relatively small in value, close to the direction of 0, especially the ASEAN countries’ Central region HM index is very close to 0. Outcome indicates from the perspective of overall trade, there is still much room for development in the future.

### 3.3 Evaluation based on explicit comparative advantage index

The Comparative Advantage Index (RCA Index) is used to measure the competitiveness of a certain product or industry in the international market. From RCA index formula if *RCA*>2.5, it indicates that the export of such commodities in the region is extremely competitive; if 2.5≥*RCA*≥1.25, it indicates that the export of such commodities in the region has strong international competitiveness; if 1.25≥*RCA*≥0.8, it indicates the export of such goods in the region which has moderate international competitiveness; if *RCA*<0.8, it indicates that the competitiveness is weak.

Table 4 demonstrate the RCA index of the top 20 commodities in the Central region from 2010 to 2017. Due to changes in the export rankings in various years, the top 20 commodities in export are selected based on the 2017 data. As it can be seen from the data in the table, among the top 20 commodities are exported from the central region, competitive commodities account for the vast majority. Luo et al. [66] mention that, the worldwide competitiveness of aquatic products assemblies of China and ASEAN are fairly dissimilar with few overlaps of strong competitive products, and there is a huge gap between the two areas in numerous kinds of products. From the perspective of commodity classification, Category 61, 62, 64, and 67 are all labor-intensive products. Since the implementation of the "Rise of Central China" strategy in the Central region, it has actively undertaken industrial transfers in the east, among which labor-intensive industries bear the brunt, and it has gradually become an important industry for foreign exchange earnings in the central region.

**Table 4:** RCA index of major export commodities in Central China from 2010 to 2017.

HS code	2010	2011	2012	2013	2014	2015	2016	2017
85	0.88	1.33	2.06	2.16	2.17	2.33	2.50	2.43
84	0.90	0.98	0.94	0.93	1.00	0.81	0.85	0.91
29	1.93	1.73	1.53	1.51	1.53	1.35	1.52	1.77
87	0.70	0.78	0.74	0.62	0.57	0.49	0.42	0.44
62	3.19	3.06	2.72	2.80	3.54	2.93	2.79	2.73
61	3.80	4.24	4.82	4.92	3.83	3.38	2.88	2.53
72	2.49	2.30	1.68	1.35	1.65	1.45	1.34	1.16
28	5.00	4.75	3.89	3.53	3.78	2.68	3.04	3.51
64	2.20	2.26	3.12	3.18	2.96	2.98	2.96	2.72
94	2.84	2.00	2.84	2.58	1.47	1.45	1.35	1.45
90	0.30	0.36	0.40	0.46	0.51	0.54	0.55	0.59
73	1.85	1.79	1.70	1.34	1.18	1.01	0.89	0.96
7	3.94	4.26	2.00	3.39	3.02	2.86	3.11	3.64
39	0.29	0.29	0.47	0.42	0.34	0.33	0.32	0.37
76	1.36	1.73	1.02	1.03	1.21	1.13	1.18	1.27
27	0.20	0.13	0.04	0.06	0.08	0.09	0.09	0.10
38	2.32	1.47	1.10	0.93	0.88	0.78	0.80	1.06
67	59.56	47.45	43.52	37.82	38.46	29.72	26.75	24.53
71	0.42	0.39	0.24	0.44	0.57	0.52	0.18	0.30
31	3.78	2.84	2.29	2.21	2.94	4.10	3.63	3.52

**Data source:** The data is calculated and compiled by Guoyan. The HS codes involved in the table are limited due to the limited space. See Table A1 in S1 Appendix.

At the provincial level, it can be seen from Table 5, the six provinces in central China have very strong or relatively high commodities in most of which are subordinated to labor-intensive products. In addition to the strong competitiveness of these commodities, the provinces also have their own competitive advantages. For example, Jiangxi and Hunan have very high competitiveness in flammable products such as explosives and fireworks. Hubei has high competitiveness in fertilizers products; Shanxi has very competitive in base metals and their parts, and Henan has very competitive in processing feathers, down and its products.

**Table 5:** RCA index of competitive export commodities of six central provinces in 2017.

Province	Extremely competitive (RCA>2.5)	Strong competitiveness (1.25<RCA<2.5)	Moderately competitive (0.8<RCA<1.25)
Jiangxi	16 (2.53), 28 (6.0), 36 (28.40), 42 (3.71), 53 (19.04), 55 (3.44), 58 (6.36), 61 (9.70), 62 (6.40), 63 (3.13), 64 (8.65), 66 (26.09), 67 (10.70), 68 (9.0), 69 (3.33), 70 (2.51), 81 (2.76), 94 (3.72), 95 (3.34)	01 (1.87), 25 (1.28), 29 (2.13), 38 (2.13), 43 (1.93), 46 (2.43), 52 (2.33), 56 (1.35), 57 (1.97), 60 (1.64), 65 (2.12), 74 (2.50), 82 (1.50), 83 (1.42), 96 (1.88)	09 (0.83), 14 (0.81), 32 (1.09), 35 (0.81), 44 (1.20), 54 (1.05), 85 (1.19)
Hubei	07 (9.07), 28 (2.74), 29 (3.18), 31 (15.17), 43 (15.22), 53 (2.97), 55 (4.04), 62 (3.37), 63 (3.48), 65 (6.35)	09 (1.57), 13 (1.73), 14 (1.84), 16 (1.80), 32 (1.43), 52 (1.79), 56 (1.81), 64 (1.71), 68 (2.08), 69 (1.32), 70 (1.41), 84 (1.27), 85 (2.07), 92 (1.27)	01 (0.97), 05 (0.89), 20 (1.15), 38 (0.90), 42 (1.17), 61 (1.12), 67 (0.99), 72 (1.20), 73 (1.08), 76 (0.82), 81 (1.21), 82 (0.99), 86 (1.16), 89 (1.20), 96 (0.88)
Hunan	05 (8.90), 13 (10.79), 16 (4.62), 28 (7.68), 36 (119.25), 42 (6.11), 43 (44.85), 53 (5.55), 64 (5.35), 67 (27.44), 69 (6.89), 78 (4.44), 81 (25.33), 82 (2.89), 86 (6.63), 91 (3.58), 96 (4.86)	01 (1.36), 07 (1.64), 09 (1.97), 20 (1.29), 24 (2.22), 35 (2.17), 46 (1.94), 52 (1.26), 58 (1.64), 63 (1.38), 71 (1.64), 73 (1.71), 85 (1.74), 95 (1.76)	02 (1.03), 12 (0.85), 29 (1.10), 41 (0.84), 61 (1.24), 62 (0.94), 65 (0.91), 66 (1.08), 76 (0.98), 83 (0.91), 94 (1.11), 48 (1.08)
Anhui	05 (7.64), 09 (3.14), 11 (2.61), 20 (2.56), 46 (27.30), 50 (3.07), 52 (2.69), 53 (8.69), 56 (3.21), 59 (3.66), 61 (2.57), 62 (4.37), 63 (4.01), 67 (12.72), 70 (3.87)	14 (1.97), 25 (2.0), 29 (2.12), 31 (1.42), 40 (1.50), 42 (2.50), 44 (1.58), 54 (2.49), 55 (2.14), 64 (1.44), 65 (1.34), 73 (1.47), 84 (2.28), 94 (2.07), 95 (2.46), 96 (1.32)	12 (1.07), 13 (1.24), 28 (0.81), 32 (1.05), 37 (0.81), 38 (1.15), 39 (0.80), 72 (0.96), 85 (0.84), 86 (1.07), 87 (0.85), 90 (1.21)
Shanxi	25 (6.57), 28 (4.11), 31 (5.11), 69 (4.05), 70 (3.11), 72 (6.11), 81 (51.44), 85 (3.08), 86 (5.67)	08 (1.48), 20 (2.41), 52 (1.75), 73 (1.45)	27 (1.09), 29 (0.90), 36 (0.90), 38 (0.89)
Henan	05 (4.44), 07 (5.63), 14 (3.31), 28 (2.68), 43 (6.83), 46 (2.93), 54 (2.54), 67 (57.97), 69 (2.80), 76 (2.84), 85 (4.27)	01 (1.48), 20 (1.96), 32 (1.62), 37 (2.24), 52 (2.43), 55 (1.32), 59 (1.42), 65 (2.01), 81 (2.20)	29 (1.03), 40 (0.94), 50 (1.17), 61 (0.88), 62 (0.91), 63 (1.21), 64 (0.90), 68 (0.99), 70 (0.84), 94 (0.88)

**Data source:** It is calculated and compiled by the data of the National Research Network. The HS codes involved in the table are limited due to the limited space.

In Table 6, the RCA index of ASEAN countries shows completely different characteristics. Some countries of ASEAN, represented by Laos, Myanmar, and Vietnam, have shown strong competitiveness in plant products such as fruits, vegetables, grains, and coffee. The countries of ASEAN particularly Indonesia and Malaysia have shown extremely strong competitiveness in animal and vegetable oils, rubber, tin and their products. In addition, Indonesia also has a strong competitiveness in commodities such as wood and wood products, and wood pulp, while Malaysia also shows strong competitiveness in base metals and their products. The ASEAN countries mainly export natural products, the exception is found in Vietnam. Cambodia has become the main destination country for the current international industrial transfer due to the abundant and cheap labor, which has led to the export of labor-intensive commodities such as textiles, shoes and hats in the region, and therefore has a strong competitive advantage in the international market. This finding is also supported by the study of Bi & Shi [54], in which they reported that Cambodia will be get more advantage for their cheap labor.

**Table 6:** RCA index of export competitive commodities of ASEAN countries in 2017.

Area	Extremely competitive	Strong competitiveness	Moderately competitive
ASEAN	14 (3.49), 15 (5.24), 16 (2.80), 40 (2.75), 80 (6.74)	03 (1.50), 08 (1.33), 09 (1.75), 11 (1.71), 17 (1.29), 21 (1.37), 44 (1.49), 46 (1.95), 55 (1.58), 61 (1.66), 62 (1.43), 64 (2.25), 75 (1.44), 85 (1.81), 92 (1.58)	10 (1.22), 18 (0.87), 19 (1.12), 20 (0.87), 24 (0.95), 25 (0.82), 27 (0.95), 29 (0.98), 33 (1.0), 34 (0.94), 38 (1.11), 39 (0.92), 42 (1.13), 43 (0.92), 47 (0.90), 52 (0.99), 54 (0.96), 67 (0.93), 71 (0.84), 74 (1.05), 78 (0.86), 84 (0.92), 90 (0.96), 99 (0.91)

**Source:** United Nations Commodity Trade Statistics Database (UN Comtrade).

Comparing the RCA index of the export commodities of the Central region and ASEAN countries, it can be found that there is a big difference in the export structure between them. Comparing the RCA index values, it is found that the overall RCA index of the Central region is higher than that of ASEAN countries which shows that the competitiveness of electrical equipment products in Central regions is higher than that of ASEAN countries. This finding shows that Cambodia and Vietnam are in the position of the competitiveness of textile, apparel, footwear and with other commodities, and they have increased this competitiveness that of the Central provinces. While ASEAN’s primary products represent the natural resource commodities which have a comparative advantage, in this regard the Central region is relatively weak.

### 3.4 Evaluation based on trade complementarity index

The Trade Complementarity Index method is used to measure the trade complementarity between the Central region and ASEAN countries.

In Table 7, the perspective of exports from the Central region and imports from ASEAN, the trade complementarity between those is constantly strengthening, from 0.89 in 2005 to 0.93 in 2011, and then to 1.10 in 2017, has been on the rise. In terms of specific countries, the trade complementarity between the Central region and Malaysia, Singapore and the Philippines has been significantly strengthened. Trade complementarity with Thailand is generally stable, and the trade complementarity index overs around 1. Trade complementarity with Vietnam fluctuates slightly. The index shows a trend of decreasing first and then increasing, but the overall remained above 1 and the ASEAN country with the strongest mutual complementarity between exports from the Central region and imports from ASEAN.

**Table 7:** Trade complementarity index between Central region and major ASEAN countries.

Country	Central region Export and ASEAN Import			ASEAN Export and Central region Import		
	2005	2011	2017	2005	2011	2017
Indonesia	1.19	0.99	1.05	2.23	1.41	0.94
Malaysia	0.78	1.02	1.19	0.75	0.74	1.25
Singapore	0.73	0.79	1.07	0.71	0.67	1.16
Philippines	0.72	0.74	1.04	0.91	1.24	1.81
Thailand	1.04	0.99	1.03	0.72	0.72	0.86
Vietnam	1.28	1.15	1.31	0.50	0.48	1.18
ASEAN	0.89	0.93	1.10	0.92	0.82	1.11

**Data source:** Calculated and compiled by Guoyan.com and the United Nations Commodity Trade Statistics Database.

From the perspective of ASEAN exports and Central region imports, the trade complementarity between those is also continuously strengthened, and the trade complementarity index has changed from less than 1 to greater than 1. This is reflected in the increasing trade complementarity with the four countries of Malaysia, Singapore, the Philippines, and Vietnam. The trade complementarity index has increased from below 1 to above 1, especially the Philippines and Vietnam perform most prominently. Trade complementarity with Thailand is relatively weak, and the trade complementarity index has remained below one.

It can be seen that the trade between the Central region and ASEAN countries has significant complementarity, and the complementarity is constantly strengthening. With continuous deepening, this kind of complementarity is getting stronger and stronger, and the degree of matching of import and export commodities is also getting higher and higher. It is true that there is a strong trade complementarity between the Central region and ASEAN, which includes both resource complementarity and industrial structure complementarity. Based on this complementarity, the Central region should adjust its industrial structure and trade direction to achieve its strengths and complement its strengths.

## 4. Empirical analysis

From the analysis of the trade relationship between the Central region and ASEAN countries, it can be seen that the trade links between those are getting closer and closer, and the complementarity of bilateral trade should exceed the competitiveness. The actual current stage of these types of bilateral trade opportunity and the countries which still have greater opportunity to be tapped but the fact is not confirmed are needed to be examined. Based on this issue, further we will use the trade gravity model to explore the trade development potential of the Central region and ASEAN countries, with a view to providing a realistic basis for the deepening of the trade cooperation between them.

### 4.1 Test and result analysis

Based on the sample data in this article as panel data, it is necessary to determine the panel data model used before empirical verification to ensure that the test results are correct. There are two ways to process panel data, one is a fixed effect model and another is a random effect model. Among the explanatory variables in this paper, the explanatory variable *DIST*_*ij*_, which measures the transportation cost by the distance from Wuhan to ASEAN countries, is fixed, a random effect model should be chosen instead of a fixed effect model. The Hausman test also has been showed no significant features, so this article finally decided to use a random effects model for empirical research.

In order to ensure the explanatory ability of the model, this paper first uses the traditional gravity model to regress the sample data, and then adds the variables *POP*_*i*_ and *POP*_*j*_ representing the population size, and the dummy variables *FTA*_*ij*_ and *Border*_*ij*_ on the basis of the traditional gravity model in turn, and the regression results of the gravity model as shown in Table 8.

**Table 8:** Regression results of extended gravity model.

Explained variable: *LnEXP*_*ij*_				
Explanatory variables	(1)	(2)	(3)	(4)
*LnGDP*_*i*_	0.9021*** (18.3728)	0.7640*** (7.7049)	0.7524*** (7.5153)	0.7513*** (7.7794)
*LnGDP*_*j*_	0.7067*** (7.7803)	0.7646*** (7.3183)	0.7242*** (6.4647)	0.7239*** (8.2437)
*LnDIST*_*ij*_	-0.5471** (-2.3033)	-0.6062** (-2.3989)	-0.5387** (-2.0285)	-0.3634** (-1.9999)
*LnPOP*_*i*_	-	5.0751 (1.5120)	4.4768 (1.3135)	4.5103 (1.3351)
*LnPOP*_*j*_	-	-0.0973 (-0.6412)	-0.0795 (-0.5091)	-0.1444 (-1.3606)
*FTA*_*ij*_	-	-	0.0845 (1.2141)	0.0941 (1.4128)
*Border*_*ij*_	-	-	-	0.9373 (1.3471)
Constant term	1.7726 (0.9992)	-49.3344 (-1.4378)	-43.3214 (-1.2440)	-44.6068 (-1.2891)
*R* ^2^	0.907512	0.9097	0.9109	0.909787
After adjustment *R*^2^	0.905516	0.9064	0.9070	0.905109

**Note**: The statistical value of t is in parentheses, ***, ** indicate the significance test at 1% and 5% respectively.

It can be seen from Table 8 that the *R*^2^ of the four regression equations and the adjusted *R*^2^ are all above 0.9, which indicates that the model has a good fitting and strong interpretation ability. But the results of the four regression equations show that the variables *LnGDP*_*i*_ and *LnGDP*_*j*_ pass the significance test at the level of 1%, the variable *LnDIST*_*ij*_ passes the significance test at the level of 5%, and the remaining variables are not significant. This shows that the variable *LnGDP*_*i*_ and the variable *LnGDP*_*j*_ and the variable *LnDIST*_*ij*_ have a higher explanatory power to the model, and the other variables have relatively weaker explanatory power. The traditional gravitational model is more suitable for the empirical research in this paper. Therefore, in order to ensure the accuracy of the results, this article decided to use the traditional gravity model with significant variables to measure the trade potential between the Central region and ASEAN countries.

The regression equation of the traditional gravity model has been obtained from the regression results from the above table and it follows:

$${InEXP}_{ij}={0.9021InGDP}_{i}+{0.7067InGDP}_{j}-{0.5471InDIST}_{ij}+1.7726$$ (i)

The regression equation of the traditional gravity model indicates that for every 1% increase in GDP of the Central region and each trading country, bilateral trade exports will increase by 0.9021% and 0.7067% respectively; for each 1% increase in transportation costs between the Central region and each trading country, and the amount of bilateral trade exports will be reduced by 0.5471%. It can be seen from this that the level of economic development and transportation costs are the most important factors affecting the level of trade development between the Central region and various trading countries, and the degree of economic development on trade is much greater than the impact of transportation costs on trade.

Further analysis of the regression results in the Central region can find:

1. The coefficients of the variables *GDP*_*i*_ and *GDP*_*j*_ are positive, and pass the significance test at 1% level, showing a highly significant characteristic, which shows that the export volume of the Central region and various trading countries is highly affected by GDP, and it grows as the GDP of both parties to the trade increases. From the point of view of the coefficient value, the coefficient value of GDP in the Central region is obviously greater than that of the GDP of the trading country. This shows that the GDP growth of the Central region has a stronger role in promoting bilateral trade than the GDP growth of the trading country.
2. The coefficient of the distance variable *DIST*_*ij*_ representing transportation costs is significantly negative, indicating that distance is the core factor affecting bilateral trade. The increase in distance means the increase in transportation costs, and the increase in transportation costs will directly lead to the increase in the export price of commodities, so that the export commodities will lose their competitive advantage in the international market, and the trade volume will be reduced. The facts also prove that the Central region has relatively higher trade volume with countries that are closer, and relatively less trade with countries that are further away.
3. The coefficient signs of the variable *POP*_*i*_ and the variable *POP*_*j*_ are inconsistent, in which the coefficient sign of the variable *POP*_*i*_ is positive and the coefficient sign of the variable *POP*_*j*_ is negative, and neither is significant. The reason for this phenomenon is that the central region’s trade with ASEAN is dominated by exports, and most of the exported products are labor-intensive products. The growth of the population has a clear role in promoting the export of labor-intensive products, so the variable *POP*_*i*_, the sign of the coefficient is positive. Exports are mainly concentrated in resource-based products. The increase in population has no obvious effect on exports, but instead promotes the supply of domestic production, so the coefficient sign of the variable *POP*_*j*_ is negative.
4. The coefficient of the dummy variable *FTA*_*ij*_ is positive but not significant, which shows that the signing of the free trade agreement has a positive effect on bilateral trade and can greatly increase the bilateral trade volume. This is evidenced by the fact that the volume of trade has increased significantly. However, the coefficient of *FTA*_*ij*_ is not significant because the bilateral trade volume between China and ASEAN has been rising before the establishment of the free trade zone. The China-Australia and China-South Korea free trade agreements are signed for a relatively short period of time, which do not fully reflect the trade promotion effect, so they are not significant in the regression results.
5. The coefficient of the dummy variable *Border*_*ij*_ is positive and passes the significance test at the level of 5%, which shows that border have a certain promotion effect on bilateral trade. This effect is mainly manifested in two aspects: first, border means closer distance and lower transportation cost; second, border can generate border trade. If the dummy variable *Border*_*ij*_ is not significant, it shows that the border does not attract the foreign trade. Since the Central region is located inland, neither along the border nor by the sea, it is less likely to produce border trade. Therefore, whether the borders are bordered has a very limited impact on the trade in the Central region.

According to the same method, the gravity models of the central provinces and trading countries can also be obtained. At this time, *EXP*_*ij*_ is the export value of the central provinces and trading countries, *GDP*_*i*_ is the economic development level of the central provinces, and *DIST*_*ij*_ is the capital cities of the central provinces to the trading countries. The distance to the capital, *POP*_*i*_ is the population of the central provinces, and the meaning of the remaining variables remains unchanged. The resulting regression equation is shown in Table 9. After a comparative analysis of the gravity model of the six central provinces, the following two characteristics can be found:

**Table 9:** Gravity model equations of central provinces.

Jiangxi	${InEXP}_{ij}={1.5767InGDP}_{i}+{0.7443InGDP}_{j}-{0.5569InDIST}_{ij}-6.1901$ (i)
Hubei	${InEXP}_{ij}={0.8954InGDP}_{i}+{0.8581InGDP}_{j}-{0.8163InDIST}_{ij}+2.6468$ (ii)
Hunan	${InEXP}_{ij}={0.7147InGDP}_{i}+{0.6958InGDP}_{j}-{0.6751InDIST}_{ij}+2.3840$ (iii)
Anhui	${InEXP}_{ij}={0.9954InGDP}_{i}+{0.6638InGDP}_{j}-{0.4212InDIST}_{ij}-1.4242$ (iv)
Shanxi	${InEXP}_{ij}={0.4162InGDP}_{i}+{0.8013InGDP}_{j}-{0.5770InDIST}_{ij}+3.0947$ (v)
Henan	${InEXP}_{ij}={1.3351InGDP}_{i}+{0.6077InGDP}_{j}-{0.3271InDIST}_{ij}-5.5971$ (vi)

Firstly, on the coefficient symbols of each explanatory variable, the gravity model equations of the six central provinces are consistent with the gravity model equations of the central region. The coefficient symbols of the variables *GDP*_*i*_ and *GDP*_*j*_ are positive, and the coefficient symbols of the variable *DIST*_*ij*_ are negative, which shows the analysis of the regression results in the Central region also applies to the central provinces. The export volume of the provinces and trading countries in the Central region is positively correlated with the GDP of both sides of the trade, and negatively correlated with the distance. This statement is in line with the findings of the studies of [16, 27, 30].

Secondly, in terms of the coefficients of variable *GDP*_*i*_ and variable *GDP*_*j*_, the coefficient value of variable *GDP*_*i*_ in the five provinces of Jiangxi, Hubei, Hunan, Anhui, and Henan should be greater than that of variable *GDP*_*j*_, that is, the coefficient of GDP of this province should be significantly larger than that of trading the coefficient value of GDP, while Shanxi Province shows the opposite characteristics. The results show that the GDP growth of the five province has a stronger role in promoting bilateral trade than the GDP growth of the trading countries. For Shanxi Province, the bilateral trade volume’s growth is more dependent on the GDP growth of trading countries, because Shanxi Province’s dependence on foreign trade is relatively low, and the proportion of imports is relatively high.

### 4.2 Trade potential in the central region

According to the trade gravity model, the various values of the Central region and ASEAN countries can be substituted into the theoretical value of the trade export between them. Comparing the actual value with the theoretical value is the trade potential index (TP index) of the Central region and ASEAN countries.


$$TP=E_{a}/E_{f}$$ (8)

Among them, *E*_*a*_ is the actual value of trade export between the Central region and ASEAN, and *E*_*f*_ is the theoretical value of trade export between the Central region and ASEAN. Drawing on the practices of the trade potential is divided into three categories: if *TP*<0.8, it means that there is still a lot of room for development on both sides, and the trade potential is huge; if 0.8≤*TP*≤1.2, which means that the trade potential of both sides has not been fully developed, and there is still some room for expansion; if *TP*>1.2, it means that the existing trade potential of both sides has been exhausted, and it is necessary to develop bilateral economic and trade cooperation through other channels [67]. The obtained measurement results according to this method are shown in Table 10.

**Table 10:** Trade potential index among Central region and ASEAN countries from 2005 to 2017.

Years	Indonesia	Malaysia	Singapore	Thailand	Vietnam	Philippines
2005	1.01	1.45	1.72	1.22	1.36	0.58
2006	0.85	1.06	2.42	1.01	1.71	0.56
2007	0.95	1.22	2.02	0.94	2.22	0.56
2008	0.98	1.28	2.17	1.00	2.11	0.60
2009	0.65	1.61	1.56	0.72	1.55	0.43
2010	0.71	1.55	1.16	0.84	2.13	0.50
2011	0.75	1.57	1.41	0.80	2.07	0.57
2012	0.87	2.47	1.80	0.97	1.16	0.62
2013	1.07	2.85	2.04	1.10	1.71	0.67
2014	1.04	2.02	2.10	1.04	2.43	0.64
2015	0.86	2.32	2.43	1.10	2.23	0.70
2016	0.68	1.82	1.86	0.97	1.95	0.67
2017	0.71	1.60	1.31	0.91	2.14	0.75

As it can be seen from Table 10, the trade potential index between the Central region and Indonesia and the Philippines is less than 0.8, indicating that the trade potential between the Central region and these two countries are huge, and there is still much room for development of bilateral trade. The trade potential index with Thailand between 0.8 and 1.2, indicating that the trade potential between the Central region and Thailand has not yet been fully tapped, and there is still room for development. While the trade potential index of Malaysia, Singapore, and Vietnam is greater than 1.2, it indicates that the Central region and these three countries’ trade potentials has reached saturation, and the available trade potential is limited. To further develop bilateral trade cooperation, new ways must be found. From the perspective of changes in the trade potential index, the characteristics of volatility changes are obvious, which has a great relationship with the economic environment in the Central region and ASEAN countries that year. For example, 2013–2015 is a golden period for the development of trade between the Central region and ASEAN countries. The trade potential index between the Central region and ASEAN countries have been reached its maximum during this period, and the potential is fully realized. Trade opportunities and a good atmosphere of cooperation are inseparable. In the past two years, due to the economic depression, the trade potential between the Central region and ASEAN countries has declined.

Table 11, shows that the trade potentials of the central provinces and ASEAN countries are generally parallel. They are manifested in the thorough development of trade potential with the three countries of Malaysia, Singapore and Vietnam. The trade potential with Indonesia and the Philippines is huge or there is room for development. This result, however, is surprising and inconsistent with the prior studies of Zhou [32] who found that Philippines has less trade potentiality. The trade potential with Thailand, the difference is relatively large. Among them, Jiangxi, Anhui, Henan and Thailand have vast trade potential, while Hubei, Hunan, Shanxi and Thailand have small trade potential. In addition, the trade potential between Henan and Malaysia is huge, the trade potential between Anhui and Singapore is really enormous, and the trade potential between Hubei and Singapore has room for development.

**Table 11:** The trade potential of provinces in Central China and ASEAN countries in 2017.

Province	Indonesia	Malaysia	Singapore	Thailand	Vietnam	Philippines
Jiangxi	0.73	1.86	1.44	0.66	1.47	0.82
Hubei	0.91	1.58	1.03	1.26	1.98	0.77
Hunan	1.07	3.02	1.87	1.27	2.74	0.44
Anhui	0.56	1.25	0.72	0.71	2.81	0.56
Shanxi	0.49	1.62	2.08	1.24	1.50	0.42
Henan	0.43	0.79	1.22	0.77	1.99	0.89

## 5. Conclusion

This paper analyses the trade relationship between the central region and ASEAN countries based on the current trade situation between them. We use the trade integration index, HM index, explicit comparative advantage index, and trade complementarity index. Then, we use the gravity model to find out the six ASEAN countries and five other major trading countries which are empirically studied in the paper. Focusing on the empirical results, the trade potentials of the six ASEAN countries are measured and we can conclude this in several important aspects.

Firstly, the trade between the central region and ASEAN has developed rapidly in the past ten years, and the bilateral trade volume has shown a rising trend. In particular, the trade with Vietnam has developed most rapidly, with an enormous increase in trade volume, making it the largest exporter in the ASEAN region in the Central Region. From the time point of view, the establishment of the ACFTA and the proposal of the “Belt and Road” strategy have greatly promoted the trade between the Central Region and ASEAN. In the future, with the deepening of the BRI strategy, the trade cooperation between the central region and ASEAN will continue to develop in both depth and breadth.

Secondly, the trade integration index and HM index hint that, the trade relationship between the Central Region and ASEAN has been significantly improved compared with before. The trade links are getting closer, especially after the establishment of the ACFTA, ASEAN has the degree of trade integration has exceeded 1, changing from alienated to close. However, in terms of the degree of trade dependence, the Central Region’s dependence on ASEAN is much greater than that of ASEAN on the Central region, and the two are characterized by asymmetry. The central region has not yet grown into a major trading area for ASEAN countries in the Chinese market, and there is still much room for development in bilateral trade.

Thirdly, the complementarity of trade between the Central Region and ASEAN countries is greater than competition. Through the calculation of the RCA index and trade complementarity index of various commodities in the Central region and ASEAN countries, it is found that the commodity trade in the Central Region and ASEAN countries is both competitive and complementary. Among the competitive commodities, textile and apparel, most labor-intensive and technology-intensive commodities such as shoes and boots and motor equipment are complementary, while complementary commodities are resource-intensive or capital-intensive commodities such as rubber, ore slag, wood pulp, cotton, animal and vegetable oils, and motors and electrical equipment. However, from the perspective of the structure of bilateral trade commodities, the import and export structure of the Central Region and ASEAN is not reasonable enough, and the overall level is relatively low.

Finally, through the trade potential between the central region and ASEAN countries, it is found that the Central Region has huge trade potential with Indonesia and the Philippines, and there is room for development with Thailand and these three countries are the focus of the Central Region in the future. The trade potential with Malaysia, Singapore and Vietnam is relatively limited, and new kinetic energy needs to be cultivated to promote the development of bilateral trade. Among the factors affecting bilateral trade, the coefficients of economic development level and distance are the most significant, and are the most important factors affecting the trade between the Central Region and ASEAN. Among them, the level of bilateral economic development plays a positive role in promoting the export trade between the Central Region and ASEAN. The bilateral distance acts as a hindrance to the reverse. Although factors such as population, free trade agreements, and borders are not significant, the free trade agreements and borders still have a positive role in promoting trade between the Central Region and ASEAN. Therefore, on the one hand, the trade cooperation between the Central Region and ASEAN should attach importance to the interconnection of infrastructure and reduce the hindrance of distance to trade. On the other hand, it should continue to promote the construction of ACFTA and actively develop the region on this basis of cooperation at different levels by adopting different cooperation strategies for ASEAN countries at different levels of development, and highlighting the advantages of the Central Region.

However, by getting the aforementioned findings, we deduce a few policy implications reported as follows:

1. The Central Region and ASEAN are highly complementary in terms of import and export commodities, but from the perspective of the number of imports and exports of various commodities, this complementarity has not been brought into full play. Therefore, policymakers should be well aware of the optimize structure of import and export commodities and improve the export level.
2. In international trade, product technology and brand advantages have always been at the top of the global value chain. Policymakers and entrepreneurs must give more priority in the strengthen of technological innovation, on the other hand they must establish their own brand, create a good image, create a good reputation, and improve product quality and diversity competition.
3. Local governments and policymakers in the Central region can use various international exhibitions and platforms to strengthen exchanges with politicians and business people from ASEAN countries attending the conference, improve communication channels with ASEAN countries, and indirectly promote the development of economic and trade relations with ASEAN countries through such official exchanges.
4. The provinces in Central region should speed up the development of rail-sea combined transport mode to build a modern, three-dimensional and efficient integrated transport channel network to improve transport efficiency, reduce transport costs, and achieve real interconnection with ASEAN countries. Policymakers need to devise plans to speed ​​up the construction of external channels and improve the level of trade facilitation.
5. The local governments should give full play to this initiative of central government, in the Central region should coordinate the work of relevant government departments such as the Foreign Affairs Office, the Department of Commerce, and the Overseas Chinese Affairs Office, simplify customs procedures, improve administrative efficiency, reduce local government control matters, and reduce administrative Examining and approving projects, reducing the handling links, creating a good environment and providing high-quality services for the majority of enterprises engaged in economic and trade cooperation as much as possible.

## Supporting information

S1 Appendix (PDF)
