# Supplementary material for: An analysis of trade cooperation: Central region in China and ASEAN
Source: PLoS One. 2021 Dec 22;16(12):e0261270. doi: 10.1371/journal.pone.0261270 (PMC8694465; doi:10.1371/journal.pone.0261270)
Supplement: S1 Appendix — (PDF) [file pone.0261270.s001.pdf]

Appendices

Table A1. Customs Import and Export HS Code

**Category 1: Live animals; animal products**<br><br>01 Live animals<br>02 Meat and food offal<br>03 Fish, crustaceans, mollusks and other aquatic invertebrates<br>04 Dairy products; eggs; natural honey; other food animal products<br>05 other animal products	**Category 2: Plant products**<br>06 Live trees and other living plants; bulbs, roots and similar products; cluster leaves for flower arrangement and decoration<br>07 food vegetables, roots and tubers<br>08 food fruits and nuts; peels of citrus fruits or melons<br>09 coffee, tea, mate tea and flavoring spices<br>10 Cereals<br>11 Milling industrial products; malt; starch; inulin; gluten<br>12 Oilseed kernels and fruits; miscellaneous kernels and fruits; industrial or medicinal plants; straw, straw and feed<br>13 shellac; gums, Resin and other plant liquids, juices<br>14 Plant materials for knitting; other plant products
**Category 3: Animal and vegetable oils, fats and their decomposition products; refined edible fats and oils; animal and vegetable waxes**<br><br>15 Animal and vegetable oils, fats and their decomposition products; refined edible fats and oils; animal and vegetable waxes	**Category 4: Food; beverages, wine and vinegar; tobacco, tobacco and tobacco substitute products**<br>16 Products of meat, fish, crustaceans, molluscs and other aquatic invertebrates<br>17 Sugar and confectionery<br>18 Cocoa and cocoa products<br>19 Products of cereals, grain flour, starch or milk; pastries<br>20 vegetables, fruits, nuts or plants Other products<br>21 Miscellaneous foods<br>22 Beverages, wine and vinegar<br>23 Residues and waste in the food industry; formulated animal feed<br>24 Tobacco, tobacco and tobacco substitute products
**Category 5: Mineral products**<br>25 Salt; sulfur; earth and stone; gypsum, lime and cement<br>26 ore, slag and ash<br>27 mineral fuel, mineral oil and its distilled products; bituminous substances; mineral wax	**Category 6: Products of the chemical industry and related industries**<br>28 Inorganic chemicals; organic and inorganic compounds of precious metals, rare earth metals, radioactive elements and their isotopes<br>29 Organic chemicals<br>30 Pharmaceuticals<br>31 Fertilizers<br>32 Tanning extracts and dye extracts; tannic acid and its derivatives; dyes, pigments and others Coloring materials; paints and varnishes; putties and other adhesives; inks, inks<br>33 essential oils and balsams; fragrance products and cosmetic toiletries<br>34 soaps, organic surfactants, detergents, lubricants, artificial waxes, modulating waxes, polish, Candles and similar products, plastic pastes, "dental wax" and dental plaster preparations<br>35 protein substances; modified starch; glue; enzymes

	36 explosives; pyrotechnic products; matches; ignition alloys; flammable materials products<br>37 Photographic and film supplies<br>38 Miscellaneous chemical products
**Category 7: Plastics and their products; rubber and its products**<br>39 Plastic and its products<br>40 Rubber and its products	**Category 8: Rawhide, leather, fur and their products; saddlery and harness; travel goods, handbags and similar containers; animal gut (except silkworm silk)**<br>41 Raw hides (except fur) and leather<br>42 Leather products; saddlery and harness; travel goods, handbags and similar containers; animal gut (except silkworm silk) products<br>43 fur, artificial fur and their products
**Category 9: Wood and wood products; charcoal; cork and cork products; straw, straw, straw or other knitting materials; baskets and wicker knitting**<br>44 Wood and wood products; charcoal<br>45 cork and cork products<br>46 straw, straw, straw or other knitting materials; baskets and wicker knitting products	**Category 10: Wood pulp and other fibrous cellulose pulp; waste and scrap of paper and cardboard; paper, cardboard and its products**<br>47 Wood pulp and other fibrous cellulose pulp; waste and scrap of paper and cardboard<br>48 Paper and cardboard; pulp, paper or cardboard products<br>49 Books, newspapers, printed drawings and other printed matter; manuscripts, typescripts and design drawings
**Category 11: Textile raw materials and textile products**<br>50 Silk<br>51 Wool, fine animal hair or coarse wool; horse hair yarn and its woven fabric<br>52 cotton<br>53 other plant textile fibers; paper yarn and its woven fabric<br>54 chemical fiber filament<br>55 chemical fiber staple fiber<br>56 batting, felt and Non-woven fabrics; special yarns; threads, ropes, cables, cables and their products<br>57 Carpets and other floor coverings of textile materials<br>58 Special woven fabrics; tufted fabrics; laces; decorative blankets; decorative tapes; embroidery<br>59 Impregnation, Coated, covered or laminated fabrics; industrial textile products<br>60 knitted fabrics and crocheted fabrics<br>61 knitted or crocheted clothing and clothing accessories<br>62 non-knitted or non-crocheted clothing and clothing accessories<br>63 Other textile products; Complete sets; old clothes and textiles; broken fabrics	**Category 12: Shoes, hats, umbrellas, sticks, whips and their parts; processed feathers and their products; artificial flowers; human hair products**<br>64 Shoes, boots, leggings and similar products and their parts<br>65 Hats and their parts<br>66 Umbrellas, parasols, canes, whips, whips and their parts<br>67 Processed feathers, down and their products; artificial flowers; human hair products
**Category 13: Products of stone, gypsum, cement, asbestos, mica and similar materials; ceramic products; glass and its products**<br>68 Products of stone, gypsum, cement, asbestos, mica and similar materials<br>69 Ceramic products<br>70 Glass and its products	**Category 14: Natural or cultured pearls, precious or semi-precious stones, precious metals, precious metals and their products; imitation jewelry; coins**<br>71 Natural or cultured pearls, precious or semi-precious stones, precious metals, precious metals and their products; imitation jewelry; coins
**Category 15: Base metals and their products**	**Category 16: Machines, mechanical appliances,**

72 Iron and steel<br>73 Iron and steel products<br>74 Copper and its products<br>75 Nickel and its products<br>76 Aluminum and its products<br>77 Lead and its products<br>78 Zinc and its products<br>79 Tin and its products<br>80 Other base metals, cerements and their products<br>81 Base metal tools, utensils, cutlery, spoons, forks and their parts<br>82 Miscellaneous products of base metals	**electrical equipment and their parts; recorders and sound players, television image and sound recording and playback equipment and their parts and accessories**<br>83 Nuclear reactors, boilers, machines, mechanical appliances and their parts<br>84 Motors, electrical equipment and their parts; recorders and sound players, television image and sound recording and playback equipment and their parts and accessories
**Category 17: Vehicles, aircraft, ships and related transportation equipment**<br>85 Railway and tram locomotives, vehicles and their parts; railway and tram track fixtures and their parts and accessories; various machinery (including electric machinery) traffic signal equipment<br>86 vehicles and their parts and accessories, but railway and tram vehicles Except<br>87 aircraft, spacecraft and parts<br>88 ships and floating structures	**Category 18: Optical, photographic, film, metrological, inspection, medical or surgical instruments and equipment, precision instruments and equipment; watches and clocks; musical instruments; parts and accessories of the above items**<br>89 Optical, photography, film, measurement, inspection, medical or surgical instruments and equipment, precision instruments and equipment; parts and accessories of the above items<br>90 watches and their parts<br>91 musical instruments and their parts and accessories
**Category 19: Weapons, ammunition and their parts and accessories**<br>92 Weapons, ammunition, parts and accessories	**Category 20: Miscellaneous products**<br>93 Furniture; bedding, mattress pads, spring mattresses, soft cushions and similar filled products; lamps and lighting fixtures not listed; illuminated signs, illuminated nameplates and similar products; mobile homes<br>94 toys, games, sporting goods and their parts, Annex<br>95 Miscellaneous products
**Category 21: Art, Collectibles and Antiquities**<br>96 Artworks, collectibles and antiquities	**Category 22: Special traded goods and unclassified**<br>97 Special transaction and unclassified

Table A2. RCA index of ASEAN countries' exports in 2017

HS Code	Brunei	Myanmar	Cambodia	Indonesia	Laos	Malaysia	Philippines	Singapore	Thailand	Vietnam
1	0.0067	0.3713	0.6357	0.2988	1.1356	0.6620	0.0242	0.0077	0.5420	0.0750
2	0.0009	0.0616	0.0001	0.0153	0.0002	0.0239	0.0203	0.0355	0.4805	0.0657
3	0.1433	7.0124	0.1422	2.8481	0.0008	0.3440	1.1397	0.1044	1.3244	4.1877
4	0.0034	0.0643	0.0067	0.3808	0.0194	0.4393	0.0611	0.1682	0.2456	0.1212
5	0.0000	0.8567	0.1107	0.2314	0.0027	0.0682	0.0563	0.0713	0.3641	0.3008
6	0.0030	0.1098	0.0002	0.0876	0.0080	0.5383	0.0737	0.0484	0.4401	0.2637
7	0.0015	16.2353	7.9332	0.1482	6.1905	0.2945	0.0877	0.0151	1.4104	0.6868
8	0.0003	2.6226	1.5629	0.8149	0.5580	0.1202	3.8166	0.0538	1.4496	4.1868
9	0.0023	0.9585	0.2971	3.9694	8.8823	0.1966	0.0175	0.2047	0.2156	6.5710
10	0.0045	16.3192	3.2725	0.0044	3.2004	0.0105	0.0063	0.0178	3.8398	1.8434
11	0.0143	0.1018	1.4489	0.3834	3.2978	0.4279	0.3252	0.1220	4.9608	3.7981
12	0.0180	3.9610	0.3010	0.2696	1.0494	0.0445	0.0585	0.0402	0.1563	0.0790
13	0.0000	0.0138	0.0977	1.3737	4.0886	0.1258	5.8054	0.1774	0.2758	0.4931
14	0.0255	6.9498	0.2868	15.2740	7.9712	5.4728	0.3168	0.3440	0.5378	1.9866
15	0.0040	0.0255	0.2451	24.0321	0.0090	11.0025	4.2805	0.1316	0.4204	0.1496
16	0.0181	0.0208	0.0002	1.9924	0.0015	0.4625	2.7833	0.0723	9.6108	3.5949
17	0.0033	21.3926	0.6566	0.4977	2.9474	0.3891	1.3446	0.0698	4.3994	0.2265
18	0.0010	0.0048	0.0143	2.4164	0.0001	2.1658	0.0794	0.6690	0.0522	0.0333
19	0.0202	0.0618	0.0749	1.4169	0.0013	1.5517	0.9162	0.9991	1.3639	0.6991
20	0.0049	0.0439	0.0318	0.4862	0.2389	0.2124	2.4728	0.1608	2.7417	0.6394
21	0.0416	0.0690	0.0261	1.5350	0.3321	1.5645	0.5717	1.1846	2.3682	0.7657
22	0.0662	0.0656	0.1604	0.1620	0.3197	0.5465	0.2896	1.0966	1.0576	0.1882
23	0.0063	0.0162	0.5585	0.8966	0.0930	0.5016	0.2703	0.1571	1.6663	0.7010
24	0.0000	1.6667	0.6020	2.5889	2.3051	0.4416	1.6618	1.1221	0.2006	0.4293
25	0.0174	0.0206	0.7006	0.5222	5.3525	0.6377	0.1131	0.0601	1.5598	1.9427
26	0.0009	0.1716	0.0001	1.9414	10.7211	0.5697	1.4913	0.0061	0.0284	0.0441
27	7.9402	2.3640	0.0003	1.9361	2.0950	1.3550	0.1338	1.1355	0.3096	0.1707

28	0.0187	0.2122	0.0001	0.7709	0.3335	0.6425	0.5656	0.1835	0.4547	0.6835
29	1.1598	0.0111	0.0547	0.8553	0.0009	0.8108	0.3752	1.9293	0.9182	0.0414
30	0.0017	0.0003	0.0138	0.1099	0.0001	0.0359	0.0245	0.5251	0.0718	0.0221
31	0.0052	0.0074	0.0000	0.5811	8.6963	0.9029	0.1397	0.0222	0.3018	0.4156
32	0.0115	0.0041	0.0016	0.7025	0.0424	0.6908	0.0960	0.8086	0.5918	0.0904
33	0.0069	0.0179	0.0126	0.5726	0.1157	0.2068	0.2304	2.3521	1.0082	0.1922
34	0.0078	0.0043	0.0024	1.8661	0.0038	1.2953	0.6033	0.7024	0.9233	0.5670
35	0.0358	0.0013	0.2667	0.1931	0.0001	0.5063	0.0606	0.5624	1.9801	0.3109
36	0.0374	0.0000	0.0002	0.2545	0.0493	0.2721	1.4886	0.6814	0.1875	0.1244
37	0.0009	0.0267	0.0065	0.0049	0.0000	1.0832	0.0229	0.4911	0.0660	0.0301
38	1.0072	0.0041	0.0082	2.1453	0.0194	1.8167	0.2728	1.4508	0.3974	0.2584
39	0.0072	0.1061	0.2599	0.4189	0.0366	1.0141	0.3218	1.1531	1.5889	0.4505
40	0.0349	1.5788	1.1192	4.3524	4.9024	3.1519	0.5390	0.5368	6.5358	1.6818
41	0.0158	0.3869	0.2020	0.3399	0.0409	0.0840	0.0318	0.1763	1.5953	1.4347
42	0.0409	1.4409	5.5688	0.6194	0.1486	0.0666	1.8298	0.8540	0.4564	3.2718
43	0.0055	0.0000	30.6598	0.0096	0.0276	0.3888	0.5303	0.0319	1.0313	1.2482
44	0.0350	2.1162	2.4378	3.0663	7.9640	2.0892	2.6546	0.0340	1.5675	1.4859
45	0.0000	0.0000	0.0032	0.0135	0.0000	0.0101	0.0049	0.0253	0.0122	0.0103
46	0.0069	0.2132	0.5321	3.2428	0.0416	0.0477	5.1015	0.0409	0.2651	7.3706
47	0.0849	0.0580	0.0590	5.3900	0.2621	0.0037	0.6214	0.4648	0.2581	0.0054
48	0.0143	0.0480	0.0593	2.4469	0.0108	0.4655	0.1799	0.4776	0.6944	0.3697
49	0.0274	0.0035	0.0359	0.0911	0.1920	0.7309	0.1591	1.0787	0.1361	0.0953
50	0.0030	0.1347	0.1187	0.0131	0.1558	0.3777	0.0010	0.0715	0.2903	3.6719
51	0.0000	0.0000	0.0001	0.0084	0.0000	0.1943	0.0001	0.0176	0.2698	0.0366
52	0.0019	0.3066	0.0355	1.4971	0.0047	0.3734	0.0181	0.0277	0.6290	3.7897
53	0.0022	0.0707	0.0011	0.2041	0.1974	0.0969	2.6685	0.0075	0.3119	0.6067
54	0.0005	0.0151	0.0330	2.1137	0.0009	0.7522	0.0672	0.2228	1.3154	1.6062
55	0.0003	0.2745	0.0519	5.9410	0.2789	0.7180	0.2353	0.1627	2.5595	1.1222
56	0.0014	0.0275	0.0872	0.7108	0.0187	0.7844	0.5510	0.1153	1.5570	1.0618
57	0.0049	0.0000	0.0132	0.5024	0.0055	0.0913	0.0083	0.0316	0.7678	0.2265
58	0.0005	0.4759	0.3454	0.4433	0.0151	0.1076	0.9484	0.0817	1.3120	0.6009

59	0.0031	0.0291	0.3524	0.5851	0.1189	0.2396	0.0456	0.2095	0.4930	1.7461
60	0.0013	0.1250	0.4649	0.3022	0.0052	0.3905	0.0262	0.1006	0.7383	1.7277
61	0.0769	2.7773	33.0951	1.7231	1.2655	0.3408	0.7027	0.1613	0.5326	4.3632
62	0.0769	10.7678	14.7881	1.8979	2.2690	0.1249	0.5030	0.1391	0.2525	4.4614
63	0.0311	0.9159	2.2230	0.4330	0.2238	0.2586	0.4748	0.0944	0.4190	1.8292
64	0.0176	2.2653	13.3426	3.6530	1.3181	0.0876	0.1498	0.1605	0.3321	8.9181
65	0.0121	0.6288	3.5857	0.4725	0.0594	0.3357	0.3644	0.1074	0.5356	2.9785
66	0.0028	0.2803	8.1465	0.0411	0.0000	0.0217	0.5256	0.0197	0.0898	0.4704
67	0.0121	0.8809	0.7026	5.7043	2.6850	0.0154	0.7920	0.0171	0.2618	0.4418
68	0.0234	0.0732	0.0572	0.3278	0.0002	0.7367	0.3453	0.1032	0.8130	0.6275
69	0.0107	0.0069	0.0062	0.6709	0.0012	0.3960	0.1703	0.0754	1.0903	0.6840
70	0.0123	0.0097	0.0082	0.4042	0.0165	1.0713	0.3117	0.1690	0.7255	1.1817
71	0.0561	0.7491	0.5239	0.8844	0.9688	0.3700	0.5268	1.2178	1.4469	0.0863
72	0.0919	1.1972	0.0279	0.9369	0.0051	0.5053	0.0766	0.2000	0.2728	0.7285
73	0.1270	0.1799	0.0982	0.3927	0.0914	0.5960	0.3211	0.3309	1.1409	0.6665
74	0.0774	4.3212	0.2078	1.4941	9.6094	1.2313	3.6546	0.4321	0.9849	0.4877
75	0.0011	0.0011	0.0000	3.4784	0.0000	2.5011	6.0168	0.9301	0.0386	0.0061
76	0.0375	0.0529	0.6013	0.3341	0.0175	1.4869	0.2625	0.1796	0.6650	0.4260
78	0.0302	4.3684	0.1706	0.3154	2.2391	2.1522	1.2098	0.4122	0.5110	0.8631
79	0.0712	0.2339	0.0706	0.1013	0.0000	1.5875	0.0845	0.1329	0.2125	0.0583
80	0.0000	0.7708	0.0014	29.3873	0.6118	8.3153	0.7468	3.8419	2.4497	0.2969
81	0.0042	0.0019	0.0004	0.0660	0.4554	0.1325	0.0772	0.2832	0.3485	0.1763
82	0.2220	0.2677	0.0039	0.1298	0.0585	0.2538	0.3635	0.6885	0.5559	0.6212
83	0.0069	0.0845	0.2070	0.1891	0.0038	0.3353	0.8690	0.2720	0.9591	0.3454
84	0.0987	0.0936	0.0422	0.2951	0.0488	0.9307	1.1265	1.1902	1.4457	0.4406
85	0.0446	0.0372	0.1723	0.3488	0.7578	2.1946	3.2924	2.3126	1.0034	2.4367
86	0.1792	0.0128	0.0003	0.0587	0.0096	0.1305	0.0141	0.1224	0.4117	0.0049
87	0.0076	0.0086	0.2812	0.4904	0.0012	0.0955	0.2085	0.1111	1.4624	0.1310
88	0.3076	0.8957	0.0021	0.0327	0.0092	0.4838	0.5163	0.9410	0.2841	0.1045
89	0.0191	2.5400	0.0027	0.1757	0.0013	0.1497	3.1840	0.1087	0.8532	0.4115

90	0.7818	0.1449	0.1839	0.1067	0.1105	1.0947	1.0786	1.4220	0.7270	1.0533
91	0.1372	0.0238	0.1644	0.0402	0.0008	0.4048	0.2111	1.5712	0.8160	0.0812
92	0.9477	0.0004	0.0092	9.1500	0.0070	1.5918	0.0250	0.1625	0.2510	0.3486
93	0.0000	0.0000	0.0034	0.0019	0.0000	0.0288	0.8017	0.0027	0.1933	0.0002
94	0.0182	0.0336	0.4101	0.7459	0.4262	0.8907	0.4373	0.0661	0.3859	2.2544
95	0.0426	0.2211	0.3604	0.4294	0.1148	0.2566	0.3730	0.2171	0.4773	0.9028
96	0.0048	0.1434	0.2354	0.7477	0.0178	0.5703	0.8268	0.2619	0.9507	0.7884
97	0.0516	0.0216	0.0297	0.0358	0.0338	0.0083	0.0273	0.6791	0.1545	0.0074
99	0.0935	0.3315	0.0855	0.0117	3.5384	0.1625	0.0000	2.7641	0.0000	0.4909

Table A3. RCA index of export commodities in Central China from 2010 to 2017

HS Code	2010	2011	2012	2013	2014	2015	2016	2017
1	1.5899	1.4815	1.3543	1.4231	1.1601	1.1783	1.3251	1.0879
2	0.5122	0.3171	0.2857	0.2949	0.3490	0.2759	0.1962	0.1499
3	0.1365	0.1436	0.1673	0.1858	0.1579	0.1072	0.0752	0.0467
4	0.4635	0.3836	0.3186	0.2600	0.2230	0.2666	0.2857	0.2409
5	4.6518	4.8505	5.4200	5.3543	4.9409	2.7398	3.2868	3.9435
6	0.0443	0.0427	0.0310	0.0286	0.0211	0.0379	0.0566	0.0562
7	3.9401	4.2639	1.9981	3.3945	3.0184	2.8636	3.1066	3.6416
8	0.3935	0.3523	0.3172	0.2788	0.3438	0.3072	0.2802	0.3263
9	1.2427	1.0231	1.0591	1.2773	1.2169	1.0912	1.3307	1.3269
10	0.1364	0.1047	0.0561	0.0457	0.0465	0.0409	0.0512	0.0490
11	1.2262	0.8988	0.9193	1.0364	0.7611	0.6447	0.5837	0.4976
12	0.6696	0.5013	0.4930	0.4998	0.4210	0.3835	0.3635	0.3506
13	1.2940	1.5772	0.7876	1.2781	1.2142	1.3730	1.5173	1.8241
14	3.5558	3.6552	3.6261	2.7348	3.0018	2.1101	2.1731	1.8906
15	0.0906	0.0676	0.0477	0.0610	0.0830	0.0737	0.0712	0.0988
16	2.5763	2.2897	2.5677	2.5077	2.4272	1.6849	1.5994	1.4778
17	0.2115	0.1883	0.0968	0.0786	0.1116	0.1254	0.0710	0.0885
18	0.0061	0.0045	0.0035	0.0021	0.0076	0.0095	0.0033	0.0046
19	0.1864	0.1559	0.1321	0.1145	0.1068	0.0972	0.1084	0.1063
20	2.3858	2.3161	2.0728	1.9319	1.6194	1.3512	1.5901	1.6490
21	0.8472	0.7863	0.6524	0.5780	0.5026	0.4012	0.3292	0.3560
22	0.0382	0.0284	0.0236	0.0185	0.0193	0.0175	0.0163	0.0217
23	0.3361	0.2978	0.2431	0.1942	0.1728	0.1443	0.1345	0.1351
24	0.7742	0.6539	0.5734	0.5223	0.4546	0.3849	0.4548	0.3878
25	5.1318	3.0165	2.0995	1.9469	1.7202	1.4503	1.7510	1.4320
26	0.0295	0.0042	0.0143	0.0092	0.0119	0.0157	0.0168	0.0250

27	0.1964	0.1256	0.0405	0.0603	0.0791	0.0881	0.0938	0.1038
28	5.0016	4.7528	3.8874	3.5268	3.7777	2.6833	3.0383	3.5123
29	1.9321	1.7255	1.5299	1.5074	1.5343	1.3500	1.5192	1.7677
30	0.1740	0.1873	0.1569	0.1524	0.1443	0.1183	0.1304	0.1286
31	3.7784	2.8403	2.2856	2.2122	2.9358	4.1004	3.6252	3.5234
32	1.0300	1.0783	0.9115	0.8035	0.8284	0.6542	0.7818	1.1636
33	0.1627	0.1504	0.1459	0.1573	0.2049	0.2107	0.1921	0.2012
34	0.4504	0.5617	0.5408	0.4905	0.4166	0.3546	0.3078	0.3154
35	1.0036	1.0863	0.9750	1.0042	0.8747	0.7388	0.6912	0.7915
36	30.0663	28.1085	25.4484	23.1383	15.8755	18.3214	18.2837	17.2071
37	1.3867	1.1440	1.0646	0.9790	0.8717	0.8044	0.8833	0.9629
38	2.3190	1.4660	1.0963	0.9261	0.8838	0.7794	0.7992	1.0567
39	0.2919	0.2902	0.4653	0.4230	0.3357	0.3349	0.3244	0.3706
40	1.6186	1.3702	1.0743	1.0665	1.0456	0.8093	0.7571	0.6894
41	0.2529	0.1801	0.1007	0.0783	0.0822	0.0866	0.0850	0.1741
42	2.7089	2.2871	3.3278	2.7798	2.1829	2.2114	2.1380	2.0372
43	6.1383	6.7307	6.8260	5.0895	8.0214	5.6345	10.8537	9.8233
44	0.9436	0.9481	0.9544	0.8608	0.8128	0.7137	0.7652	0.6975
45	0.1210	0.1218	0.0517	0.0345	0.0356	0.0742	0.0531	0.0542
46	20.6315	21.3368	19.7533	13.9670	9.1135	7.8136	6.4210	6.4328
47	0.1763	0.2233	0.1587	0.1062	0.0857	0.0712	0.0548	0.0709
48	0.4892	0.4947	0.6320	0.5641	0.4515	0.5608	0.4885	0.4826
49	0.1203	0.1135	0.2654	0.2341	0.1752	0.1899	0.1619	0.1731
50	2.1226	1.9595	1.6722	2.2188	1.7756	0.9164	1.3204	1.0162
51	0.4855	0.4479	0.3920	0.4229	0.2415	0.3858	0.2124	0.1445
52	2.0277	2.0196	2.0060	2.6619	2.3481	2.1544	2.1921	2.1663
53	8.2513	8.4278	8.6656	13.2306	13.0967	11.4926	5.2811	5.6331
54	2.1921	1.9683	1.5648	1.4357	1.4025	1.2390	1.3908	1.5074
55	2.8893	3.0735	2.9824	2.6371	2.2970	1.7938	1.9776	2.0893

56	1.2335	1.2062	1.1369	1.3337	1.1390	1.1987	1.1379	1.2055
57	1.0068	0.8227	0.7401	0.6253	0.5717	0.4496	0.4302	0.4869
58	1.8552	2.6699	1.4384	1.4587	1.4534	1.0490	1.1371	1.4438
59	3.3514	3.6565	2.0308	1.7242	1.4900	1.1824	1.2382	1.2237
60	0.3324	0.3279	0.4019	0.3833	0.4870	0.4561	0.3570	0.4717
61	3.7979	4.2364	4.8169	4.9243	3.8263	3.3803	2.8837	2.5317
62	3.1928	3.0576	2.7159	2.8002	3.5400	2.9321	2.7950	2.7279
63	3.6225	3.0665	2.9165	2.6200	2.5636	2.2886	2.1997	2.3270
64	2.1954	2.2605	3.1189	3.1842	2.9603	2.9776	2.9611	2.7151
65	3.0177	2.3570	2.5925	2.2449	2.2551	2.2657	2.1586	2.3786
66	12.9078	8.0825	7.5625	6.3977	4.2386	3.9515	3.4817	4.3146
67	59.5645	47.4468	43.5201	37.8203	38.4574	29.7189	26.7542	24.5272
68	1.1104	1.0158	1.3946	2.2877	2.1072	2.8696	3.0693	2.2479
69	6.6806	6.1468	5.5672	4.6563	3.7205	3.2194	3.4308	2.7144
70	1.9169	2.0117	2.0763	1.9422	1.6993	1.7446	1.8551	1.8946
71	0.4248	0.3918	0.2414	0.4395	0.5674	0.5234	0.1829	0.2985
72	2.4884	2.2956	1.6787	1.3473	1.6539	1.4499	1.3400	1.1607
73	1.8474	1.7923	1.7018	1.3377	1.1752	1.0122	0.8947	0.9628
74	0.6582	0.7497	0.5991	1.0332	0.9073	0.6001	0.5290	0.6280
75	0.0751	0.0815	0.0843	0.0415	0.0385	0.0285	0.0378	0.0258
76	1.3621	1.7267	1.0154	1.0339	1.2094	1.1301	1.1779	1.2709
78	2.0876	0.9068	0.2559	1.8479	1.9192	1.9157	0.6982	0.5118
79	0.1016	0.0185	0.0260	0.0511	0.0530	0.0601	0.0445	0.0182
80	0.0018	0.0002	0.0006	0.0011	0.0035	0.0075	0.2432	0.0153
81	20.1982	16.0723	11.8096	11.7955	11.0933	8.8396	9.5590	8.4472
82	0.9430	0.9650	1.2936	1.2139	1.0340	0.8531	0.8196	0.9498
83	0.3895	0.2834	0.9052	0.7318	0.5234	0.6360	0.4037	0.5342
84	0.9003	0.9793	0.9410	0.9268	0.9992	0.8132	0.8494	0.9111
85	0.8822	1.3316	2.0572	2.1573	2.1729	2.3282	2.5022	2.4301
86	2.1637	3.6206	4.5932	2.0134	2.3619	4.4760	2.0800	1.7190

87	0.7015	0.7772	0.7377	0.6199	0.5719	0.4933	0.4172	0.4376
88	0.0309	0.0267	0.0099	0.0139	0.0198	0.0063	0.0120	0.0078
89	1.7771	1.5295	1.2623	1.0777	0.5280	0.2777	0.3400	0.3817
90	0.2995	0.3581	0.4009	0.4589	0.5064	0.5445	0.5527	0.5931
91	0.2554	0.0978	0.1900	0.2061	0.1515	0.1574	0.1867	0.4989
92	0.6526	0.6333	0.8544	0.7967	0.4875	0.4439	0.4038	0.3622
94	2.8417	1.9984	2.8448	2.5784	1.4684	1.4536	1.3550	1.4483
95	1.6916	3.3867	2.3886	2.3908	1.6285	1.3688	1.3047	1.3083
96	2.3794	2.5525	2.1380	1.5229	1.3722	1.2027	1.2738	1.4209

Table A4. Trade complementarity index of exports of central China and imports of six ASEAN countries in 2017

HS Code	Indonesia	Malaysia	Philippines	Singapore	Thailand	Vietnam
1	2.8095	0.2737	0.0569	0.4967	0.3821	1.4096
2	0.0800	0.1091	0.2444	0.0590	0.0126	0.0379
3	0.0128	0.0302	0.0384	0.0180	0.1036	0.0433
4	0.2909	0.2209	0.4628	0.1504	0.1490	0.1241
5	5.5019	1.0112	2.6898	0.8619	2.4192	8.6864
6	0.0018	0.0030	0.0011	0.0139	0.0074	0.0258
7	4.6715	4.2237	1.0200	1.4452	3.2322	2.6751
8	0.3311	0.1697	0.1587	0.0942	0.1959	0.7248
9	0.7414	1.2520	0.4332	0.4588	0.8035	0.4100
10	0.1978	0.0580	0.1387	0.0067	0.0229	0.0958
11	0.8835	0.9600	1.0907	0.2144	0.5750	0.5662
12	0.6184	0.1826	0.1574	0.0294	0.3822	0.2262
13	1.8632	1.2440	1.7214	0.7441	2.0495	1.4726
14	0.3213	0.5357	0.2231	1.6594	4.1703	0.3963
15	0.0212	0.1441	0.1933	0.0574	0.0262	0.0629
16	0.1424	0.4365	0.2850	0.7907	0.6058	0.0783
17	0.5206	0.1705	0.1292	0.0273	0.0278	0.0441
18	0.0066	0.0087	0.0030	0.0025	0.0014	0.0004
19	0.0557	0.1008	0.1087	0.0418	0.0694	0.0703
20	0.6012	0.8926	1.4516	0.6282	0.6205	0.1744
21	0.4024	0.4418	1.2764	0.2013	0.3406	0.2942
22	0.0026	0.0108	0.0186	0.0266	0.0054	0.0033
23	0.6063	0.1818	0.4549	0.0105	0.3102	0.4901
24	0.6027	0.1853	0.3809	0.3966	0.1481	0.1913
25	5.5673	1.0923	2.7330	1.4534	0.6815	0.8080
26	0.0122	0.0184	0.0404	0.0004	0.0023	0.0040
27	0.1615	0.1159	0.1020	0.2005	0.1276	0.0459
28	6.0544	6.1926	3.2261	1.8387	4.2132	2.9204
29	2.7953	1.4249	0.9213	1.4382	1.4647	1.1389
30	0.0220	0.0267	0.0674	0.0310	0.0412	0.0586
31	14.4757	5.2973	6.4220	0.0473	7.8922	5.9548
32	2.1420	1.1138	1.1472	0.8926	1.6833	1.7964
33	0.1771	0.1422	0.2167	0.3118	0.2093	0.0902
34	0.3126	0.2978	0.3698	0.1976	0.3265	0.2761
35	1.3363	0.7099	1.3743	0.5260	0.6928	1.3990
36	42.1847	7.7232	16.9851	5.3739	15.0472	3.6835
37	0.4683	0.6536	0.5599	1.3393	0.7293	0.5301
38	1.2551	1.4629	1.0821	1.4474	1.6408	1.2945
39	0.5083	0.4266	0.3476	0.2523	0.4167	0.6641

40	0.7880	1.2626	0.4249	0.2683	0.7222	0.6708
41	0.3156	0.0585	0.1490	0.0270	0.3690	0.9322
42	1.0126	1.2614	0.8709	2.6454	1.2877	0.3194
43	1.0789	5.8351	2.3889	0.6229	20.8457	31.7353
44	0.2811	0.3038	0.5850	0.1125	0.2046	0.7768
45	0.0151	0.0073	0.0093	0.0035	0.0042	0.0019
46	0.4563	2.4649	0.7128	0.9740	2.0354	3.6187
47	0.2569	0.0221	0.0226	0.0144	0.0862	0.0546
48	0.4406	0.5041	0.6069	0.2714	0.3818	0.5706
49	0.0897	0.0840	0.1613	0.1006	0.0776	0.0782
50	0.2588	0.6662	0.2106	0.1452	0.4563	3.0315
51	0.1313	0.0296	0.0240	0.0041	0.0871	0.2160
52	10.6146	1.7787	1.2491	0.0859	2.7120	14.5449
53	6.5272	0.6611	5.1799	0.1527	1.6474	8.6609
54	5.7825	1.3584	1.1437	0.1257	2.1675	7.4291
55	8.1160	0.8027	2.0253	0.4182	1.4961	14.4157
56	2.6431	0.9237	0.9340	0.3406	1.0401	3.2009
57	0.1930	0.2992	0.1393	0.1184	0.1405	0.1550
58	4.8681	0.5729	3.3107	0.1941	1.8222	11.6775
59	3.4701	0.8718	1.2243	0.4321	2.2415	5.0318
60	2.3290	0.2087	0.8939	0.0743	0.5440	6.2443
61	0.3323	1.0347	0.4288	0.8601	0.4494	0.2705
62	0.4465	0.8548	0.5237	0.8535	0.6342	0.2661
63	0.4875	1.7907	0.9365	0.5063	0.9577	0.3589
64	1.2706	1.1601	1.1013	0.7874	0.6320	1.5005
65	0.8242	0.6880	0.8165	0.5727	0.7888	0.3546
66	4.4133	1.7796	4.0456	0.8131	2.8438	1.0836
67	36.3902	7.8756	5.1477	1.8570	7.3197	17.4833
68	2.7517	1.2273	2.1166	1.4370	1.0931	1.5710
69	3.3913	1.7556	5.4711	1.0052	3.3311	1.6607
70	1.1645	2.0911	1.2900	1.1615	1.9385	2.2979
71	0.0531	0.1838	0.0128	0.4978	0.5492	0.0194
72	2.8911	1.4452	2.0741	0.3947	2.5441	2.7034
73	0.9386	0.9005	0.9264	0.5064	1.8703	0.8648
74	0.5947	1.3194	0.5204	0.2896	1.3081	0.8649
75	0.0063	0.0286	0.0057	0.0246	0.0083	0.0073
76	1.4583	1.7227	0.6097	0.4313	1.9793	1.6237
78	1.2251	0.4401	1.1975	0.0256	1.2464	1.7334
79	0.0430	0.0354	0.0057	0.0044	0.0281	0.0390
80	0.0041	0.0460	0.0186	0.0580	0.0261	0.0170
81	4.6823	2.6147	1.1051	5.1836	3.7785	1.7634
82	0.9595	0.6783	0.8816	0.7154	2.0081	0.6712
83	0.5842	0.3341	0.5102	0.1916	0.4178	0.5234

84	0.9663	0.8887	1.0053	1.0466	0.9226	0.8093
85	1.6406	4.4150	3.5562	4.4731	2.9574	4.6859
86	1.1724	0.5384	0.2951	0.5789	1.1089	0.4077
87	0.2089	0.1496	0.4595	0.0811	0.2171	0.1148
88	0.0032	0.0096	0.0110	0.0139	0.0099	0.0008
89	0.6502	0.4715	0.1411	0.0839	0.5711	0.0667
90	0.2755	0.5281	0.3486	0.6320	0.4590	0.7247
91	0.1501	0.5103	0.2811	0.9996	0.5313	0.0875
92	0.7583	0.2947	0.1564	0.1877	0.1810	0.1265
94	7.2364	0.6065	0.6616	0.3937	0.5106	0.3310
95	0.5222	0.5593	0.7404	0.4284	0.2981	0.2160
96	0.3955	0.9505	2.0193	0.5203	0.9635	2.1353
97	0.0011	0.0001	0.0000	0.0016	0.0002	0.0000
